# Proteomic Profiling of Small-Cell Lung Cancer: A Systematic Review

**DOI:** 10.3390/cancers15205005

**Published:** 2023-10-16

**Authors:** Amgad Mohamed Elshoeibi, Basel Elsayed, Muhammad Zain Kaleem, Mohamed Ragab Elhadary, Mohannad Natheef Abu-Haweeleh, Yunes Haithm, Hubert Krzyslak, Semir Vranic, Shona Pedersen

**Affiliations:** 1 College of Medicine, QU Health, Qatar University, Doha 2713, Qatarma1908120@qu.edu.qa (M.N.A.-H.); svranic@qu.edu.qa (S.V.); 2 Department of Clinical Biochemistry, Aalborg University Hospital, 9000 Aalborg, Denmark

**Keywords:** small-cell lung cancer, large cell neuroendocrine carcinoma, proteomics, diagnostics and prognostic biomarkers, neuroendocrine tumors

## Abstract

**Simple Summary:**

Our study centers on refining the diagnosis of small-cell lung cancer (SCLC), a unique subtype with distinct therapeutic implications compared to other lung cancers. Our primary goal is the identification of specific differentially expressed proteins in SCLC as opposed to healthy lung tissue. Additionally, we aim to discern the protein expression of SCLC from large cell neuroendocrine carcinoma (LCNEC), a closely related entity. This research has the potential to enhance our understanding of these intricate lung cancers, potentially transforming the landscape of detection and tailored treatment strategies.

**Abstract:**

The accurate diagnosis of small-cell lung cancer (SCLC) is crucial, as treatment strategies differ from those of other lung cancers. This systematic review aims to identify proteins differentially expressed in SCLC compared to normal lung tissue, evaluating their potential utility in diagnosing and prognosing the disease. Additionally, the study identifies proteins differentially expressed between SCLC and large cell neuroendocrine carcinoma (LCNEC), aiming to discover biomarkers distinguishing between these two subtypes of neuroendocrine lung cancers. Following the Preferred Reporting Items for Systematic Reviews and Meta-Analyses (PRISMA) guidelines, a comprehensive search was conducted across PubMed/MEDLINE, Scopus, Embase, and Web of Science databases. Studies reporting proteomics information and confirming SCLC and/or LCNEC through histopathological and/or cytopathological examination were included, while review articles, non-original articles, and studies based on animal samples or cell lines were excluded. The initial search yielded 1705 articles, and after deduplication and screening, 16 articles were deemed eligible. These studies revealed 117 unique proteins significantly differentially expressed in SCLC compared to normal lung tissue, along with 37 unique proteins differentially expressed between SCLC and LCNEC. In conclusion, this review highlights the potential of proteomics technology in identifying novel biomarkers for diagnosing SCLC, predicting its prognosis, and distinguishing it from LCNEC.

## 1. Introduction

Lung cancer remains the leading cause of cancer-related mortality worldwide, with two main types in humans: non-small-cell lung cancer (NSCLC) and small-cell lung cancer (SCLC), with the latter accounting for approximately 15% of all cases of lung cancer. Compared with NSCLC, SCLC typically exhibits a rapid rate of cell division, a higher growth fraction, and an earlier onset of metastases to other organs such as the brain, liver, adrenals, and bones [1,2]. According to the WHO, SCLC can be classified into pure SCLC, which accounts for most cases, or combined SCLC and LNEC [2,3]. Due to its aggressive course, SCLC is often diagnosed at an advanced stage, where treatment options are limited to chemotherapy and radiation rather than surgical removal [4].

SCLC and large cell neuroendocrine carcinoma (LCNEC) are two types of aggressive lung cancer sharing several common morphological and molecular characteristics. They are both high-grade neuroendocrine cancers with similar histological features, including a high mitotic rate, extensive necrosis, and frequent expression of neuroendocrine markers such as synaptophysin, chromogranin-A, and CD56/NCAM (neural cell adhesion molecule) [5,6,7,8,9]. CD56 is a particularly useful biomarker as it is highly sensitive, staining >90% of SCLC/LCNEC, while synaptophysin and chromogranin-A tend to be positive in ~50–60% of cases. Its specificity is lower as it can stain squamous cells, large cells (non-neuroendocrine), and adenocarcinomas in ~10% of cases [9]. Both SCLC and LCNEC are also positive for thyroid transcription factor 1 (TTF-1) in >80% of cases. Taken together, currently available immunohistochemical biomarkers have limited utility in discriminating SCLC from LCNEC but can be very helpful in differentiating pulmonary neuroendocrine carcinomas from other lung neoplasms and metastases to the lung [3,10].

SCLC consistently exhibits mutations in the *TP53* and *RB1* genes. However, it is a molecularly heterogeneous neoplasm with at least four different gene expression subtypes, two of which harbor *ASCL1* and *NEUROD1* alterations, whereas the remaining two have genomic aberrations of the *POU2F3* and *YAP1* genes. A subset of LCNEC also harbors mutations of *TP53* and *RB1* genes (similar to SCLC), while small proportions of LCNEC may have *MEN1* mutations as well as *KRAS* and *STK11/KEAP1* mutations, typically seen in NSCLC and not SCLC. However, these molecular genetic biomarkers are rarely utilized for diagnostic purposes, in addition to their limited predictive value in the precision oncology of lung cancer [3].

Due to these overlapping features, distinguishing between SCLC and LCNEC can be challenging, and misdiagnosis can occur. Therefore, the most recent (2021) WHO classification of lung tumors acknowledges this difficulty in recognizing a distinct entity called “combined SCLC and LCNEC” [3]. Accurate classification is critical, as the treatment strategies for these two types of cancer differ [11]. Therefore, the diagnostic criteria for SCLC and LCNEC should be clearly defined, and a multidisciplinary approach, including histological, immunohistochemical, and molecular analyses, should be used to differentiate between them [12].

Over the last two decades, several proteomics techniques have emerged, with mass spectrometry (MS) being one of the most powerful technologies for quantitative proteomics. Utilizing statistically robust algorithms, a high-throughput method permits the identification and quantification of a wide variety of peptides and proteins with exceptional sensitivity. In recent years, MS-based proteomics has been combined with other methodologies to enhance the precision and reproducibility of results. [13]. Besides label-free quantitation, stable isotope labeling has emerged as a conventional method in proteomics research. Recently introduced, super-stable isotope labeling with amino acids in cell culture (SILAC) offers a more powerful quantification method for human tissue samples [14].

Since the 1980s, considerable research has been devoted to identifying factors that could predict treatment response and survival in patients with SCLC. Many studies have explored a range of clinical, laboratory, and genetic markers to distinguish the subgroups of SCLC patients resistant to chemotherapy. Unfortunately, only a limited number of prognostic variables have gained widespread acceptance as reliable predictors for certain chemotherapy patients [14]. Furthermore, there is a scarcity of thorough research into SCLC diagnostic and early prognostic markers [15]. Consequently, this systematic review aims to identify proteins that exhibit differential expression in SCLC and evaluate their potential utility in diagnosing the disease. In addition, the review seeks to explore proteins that display distinct expression patterns between SCLC and LCNEC to identify potential biomarkers capable of differentiating the two neuroendocrine lung cancer subtypes.

## 2. Materials and Methods

### 2.1. Protocol and Registration

For this systematic review, we adhered to the Preferred Reporting Items for Systematic Reviews and Meta-Analyses (PRISMA) guideline, as indicated by the PRISMA checklist found in Table S1 in the Supplementary Materials. Furthermore, we registered the protocol with the International Prospective Register of Systematic Reviews (PROSPERO) online database under CRD42022368038.

### 2.2. Search Strategy

We developed a comprehensive search strategy for SCLC and proteomics using the PubMed/MEDLINE database. Our search strategy included Medical Subject Headings (MeSH) terms such as “Small Cell Lung Carcinoma [Mesh]” and “Proteomics [Mesh],” as well as free text terms like “small cell lung cancer/carcinoma,” “SCLC,” “large cell neuroendocrine cancer/carcinoma,” “LCNEC,” and “Proteomics” with related keywords. This approach was intended to ensure a broad search strategy for identifying relevant articles. We applied no restrictions concerning language or date, and the search was confined to studies involving human subjects. We employed the Polyglot translator to adapt our search strategy to the Scopus, Embase, and Web of Science databases [16]. The complete search strategy for each database is detailed in the Supplementary Materials. Finally, duplicates were removed using EndNote X9, and the resulting studies were screened for relevance.

### 2.3. Eligibility Criteria

We included all studies that reported proteomics information and confirmed SCLC and/or LCNEC based on histopathological and/or cytopathological examination. Eligibility criteria were applied in both the title abstract screening and the full text screening. There were no limitations placed on the study’s design. We excluded reviews, non-original studies based on animal samples or cell lines, and articles that did not report SCLC or LCNEC-related proteomics information. In cases where multiple articles utilized the same data, we only included the most recent or comprehensive article.

### 2.4. Study Selection and Screening

After applying our search strategy to the selected databases and removing duplicates with EndNote, the remaining articles were further screened using the Rayyan platform [17]. Two reviewers independently evaluated the titles and abstracts of the articles, and conflicts in screening were resolved by mutual agreement. The eligible papers were then retrieved and subjected to a second independent evaluation, with any disagreements settled through team deliberations.

### 2.5. Data Extraction

The following data were extracted from each study: last name of the first author, year of publication, tumor classifications, sample size, sample type (serum, urine, exosomes, tissue samples, etc.), mass spectrometry utilized, type of proteomic analysis (structural, functional, or expressional), proteins identified, and proteomic conclusion (prognostic, diagnostic, or predictive). In addition, the identified proteins were classified as over- and under-expressed. Two researchers independently evaluated and extracted data from eligible studies. In cases of disagreement, a third member was consulted. Finally, all team members agreed during a meeting to determine whether a study should be included in the systematic review.

### 2.6. Quality of Studies

The QUADOMICS methodology criteria were utilized to appraise the quality of the included studies, providing a specific evaluation tool for “-omics”-based diagnostic research [18]. This tool was adapted from the QUADAS scale, frequently used to assess the quality of systematic reviews of diagnostic accuracy studies, and considers the technical peculiarities inherent in omics methodologies [19]. The quality of the qualified research was independently evaluated by two investigators using the 16-item QUADOMICS scale, and a third member was involved when disagreements arose.

### 2.7. Outcomes

Our main objective for this systematic review was to examine the proteomic profiling of patients with SCLC. Additionally, we aimed to compare SCLC’s proteomic profiling with LCNEC as a secondary objective.

## 3. Results

### 3.1. Study Selection

**Figure 1:**
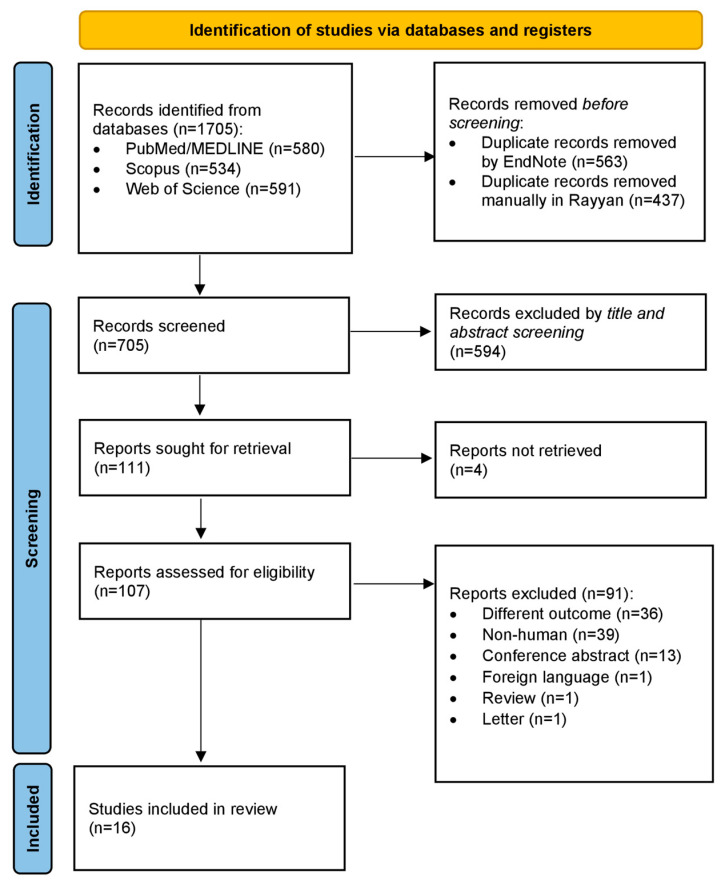
PRISMA flow-chart for the systematic review.

As depicted by the PRISMA flow chart in Figure 1, the search strategy process identified 1705 articles from PubMed/MEDLINE, Scopus, and Web of Science. These articles were imported into EndNote, where 563 duplicates were removed. The remaining 1142 articles were transferred to Rayyan, and 437 duplicate articles were manually detected and removed. This resulted in 705 unique articles, which underwent title and abstract screening, eliminating 594 articles. Four of the remaining 111 articles were unobtainable, leaving 107 for full-text screening. After reviewing these articles, 91 were excluded for one of the reasons described in the PRISMA flow chart and described in Table S2. Ultimately, 16 articles met the selection criteria and were included in our final study.

### 3.2. Study Characteristics and Data Collection

Table 1 and Table 2 present the characteristics and data collected from the studies included in this study. These articles were published between 2004 and 2022. Of the sixteen studies, the majority were conducted in Korea and the USA (four articles each), followed by Japan and China (three articles each), and one each from Hungary and Denmark. The total sample sizes ranged from 7 to 241 patients, with the sample sizes for SCLC specimens ranging from 4 to 72. The studies utilized various types of specimens, including formalin-fixed paraffin-embedded (FFPE) tissue specimens (six studies), serum samples (six studies, one of which also incorporated urine samples), urine samples exclusively (one study), and the remaining three studies utilized plasma, lung bronchoscopic biopsy, plasma-derived microvesicles, exosomes, or bronchoalveolar lavage (BAL). Regarding the proteomic method, only one study employed targeted mass spectrometry (LC/MRM-MS). The rest utilized untargeted approaches such as MALDI-TOF-MS, LC-MS/MS, SELDI-TOF-MS, LC-ESI-MS/MS, Nano LC-MS/MS, nanoUHPLC-MS(MS), and ES-MS-MS.

### 3.3. Quality Assessment

The quality assessment analysis included various studies that had different levels of safeguards. These safeguards ranged from 4 to 14 out of 16, with an average of approximately eight per study. Most studies showed a good implementation of safeguard items related to the description of selection criteria, sample type, procedures and timing of biological sample collection, handling, pre-analytical procedures, and the execution of the index test. However, there was a lack of implementation of items related to ensuring a short time gap between the reference and index tests to maintain an unchanged target condition, using the same reference standard for all patients irrespective of index test results, independent interpretation of the reference and index test results, using clinical data similar to that available in real-world practice, reporting of uninterpretable/intermediate test results, and avoiding overfitting. The remaining four items were implemented by around half of the 16 studies. Complete responses to all quality assessment items included in the QUADROMICS tool are presented in Table S3 in the Supplementary Materials.

### 3.4. Proteomic Findings in SCLC Compared with Controls

Among the 16 studies incorporated into our review, 13 explored the differences in protein expression between SCLC and healthy control subjects (Table 1). After analyzing the data, 117 unique proteins that exhibit differential expression between SCLC and controls were identified. However, when we cross-referenced these findings between the studies, we discovered that only five of these proteins were common in at least two studies. These proteins include haptoglobin (HPT), Coactosin-like protein 1 (COTL-1), hemoglobin alpha (HBA), hemoglobin beta (HBB), and desmoplakin (DESP). Shah et al. and Pedersen et al. found that HPT expression was increased in SCLC patients compared to healthy controls [28,29]. Additionally, Bharti et al. found that the alpha subunit of HP (HPA) was upregulated in SCLC compared to healthy controls. At the same time, Kang et al. reported an upregulation of the beta subunit (HPB) [21,25]. In addition, COTL-1 was identified as an upregulated protein in SCLC by Fahrmann et al. and Hye-Cheol et al. [22,24]. On the contrary, HBA and HBB subunits were downregulated proteins in SCLC identified by both Pedersen et al. and Sugar et al. [28,30]. Finally, DESP emerged as another common protein found in both the studies by Sugar et al. and Zhang et al. However, the results of these papers were inconsistent, with Sugar et al. indicating an upregulation of the protein, whereas Zhang et al. observed a downregulation in SCLC [30,31].

### 3.5. Differential Proteomic Findings between SCLC and LCNEC

The remaining three studies investigated proteins with differential expression between SCLC and LCNEC (summarized in Table 2). Across these studies, 37 unique proteins were found to exhibit differential expression between SCLC and LCNEC. Upon cross-referencing these proteins between the three studies, beta enolase (ENOB), brain acid soluble protein 1 (BASP-1), aldehyde dehydrogenase 1 family member A1 (AL1A1), and Secretagogin (SEGN) were found by at least two of the three studies. BASP-1 was identified as being upregulated in SCLC compared with LCNEC by Nomura et al. and Fukuda et al. The same two studies also identified AL1A1 and ENOB as downregulated in SCLC compared with LCNEC [33,35]. Finally, Nakamura et al. and Nomura et al. identified SEGN as a differentially expressed protein between SCLC and LCNEC [34,35]. However, their results were contradictory; the latter study observed the protein to be upregulated in SCLC, whereas the former reported it as downregulated.

## 4. Discussion

In this study, we aimed to identify protein biomarker candidates that could aid in the diagnosis and prognosis of SCLC and differentiate it from healthy tissue and LCNEC. To achieve this, we conducted a systematic review using the PRISMA guidelines. Our search strategy was comprehensive, without any language or time frame limitations. A total of 16 studies were included in our review, which utilized various proteomic techniques, including targeted and untargeted mass spectrometry approaches. Our review revealed differential expression of several proteins, including upregulation of HPT and COTL-1 and downregulation of HBA and HBB in SCLC tissue compared with healthy controls across multiple studies. Notably, DESP was found to be upregulated by one study and downregulated by another, indicating inconsistent results. We hypothesize that this inconsistency may be attributed to differences in the sample preparation and analytical techniques applied in the two studies. Specifically, Sugar et al. used FFPE human tissue samples and the nanoUHPLC-MS/MS proteomic technique, while Zhang et al. used urine samples and LC-MS/MS analysis [30,31]. These differences in sample preparation and analysis techniques could account for the observed heterogeneity in the results.

Haptoglobin is a protein synthesized in the liver and is known for binding and sequestering free hemoglobin, preventing oxidative damage. While haptoglobin is not traditionally considered a direct player in cancer signaling pathways, studies have shown that acute phase proteins, including haptoglobin, are linked to cancer, particularly in the context of inflammation and tumor microenvironment, including those with small-cell lung cancer (SCLC) [36,37,38]. Altered glycosylation of the β chain of haptoglobin is a common theme in these studies, with increases in specific glycans associated with cancer progression [39]. In other cancers, such as breast cancer, the upregulation of haptoglobin has been associated with increased tumor growth and metastasis [40]. COTL-1 is an F-actin-binding protein mainly expressed in the placenta, lungs, and kidneys [41]. It activates 5-lipoxygenase, which is involved in inflammatory diseases, cancer development, and cell survival [42].

Additionally, actin can inhibit 5-lipoxygenase by binding to its metabolites [43]. The findings suggest that COTL-1 expression may play a central role in SCLC by interacting with actin and 5-lipoxygenase. Research on the role of COTL-1 in human cancer is currently limited, but a recent study comparing epithelial breast cancer cells to their mesenchymal counterparts in mice, which also examined some human breast cancer cell lines, has shed some light on potential mechanisms. The study revealed two mechanisms that could explain the role of COTL-1 in cancer. First, when COTL-1 was re-expressed in mesenchymal mouse breast cancer cells (FE 1.2) and human MCF7 breast cancer cells, it led to the activation of the growth-suppressor gene interleukin-24 (IL-24). This, independently of p53, resulted in an upregulation of tumor-suppressor genes like p53 apoptosis effector related to PMP-22 (PERP) and p21cip1. Second, the study found that the overexpression of COTL-1 amplified the growth-suppressive effects of transforming growth factor-β1 (TGFβ1). This led to the downregulation of TGFβ-responsive genes, including vascular growth factor A/B (VEGFA/VEGFB), hypoxia-inducing factor 1α (HIF-1α), and thrombospondin 1 (TSP1), all of which play crucial roles in various aspects of cancer progression such as angiogenesis, invasion, and metastasis. While these findings are promising, further research in human cancer contexts is necessary to confirm and expand upon these observations [44]. The included study by Pedersen et al. found that patients with SCLC had downregulated levels of blood hemoglobin markers, which is opposite to the upregulated levels observed in NSCLC patients [28,45,46]. A recent report suggested that the decreased hemoglobin-to-red blood cell distribution width ratio in both SCLC and NSCLC patients is associated with poor prognosis, potentially due to increased hypoxic cells contributing to an aggressive tumor phenotype [47]. Pedersen et al.’s findings support the idea that oxidative stress may be a factor in the development or progression of SCLC [28]. Yang et al. suggest that DESP functions as a tumor suppressor in lung cancer by inhibiting the Wnt/β-catenin signaling pathway [48]. The data indicate that DESP is inactivated by an epigenetic mechanism in lung cancer and has the potential to increase the sensitivity to anticancer drug-induced apoptosis [48].

The differential expression of proteins between SCLC and LCNEC was investigated in three studies. Nomura et al. and Fukuda et al. reported an upregulation of BASP-1 in SCLC compared with LCNEC and a downregulation of AL1A1. BASP-1 is a protein encoded by the BASP1 gene and has been found to be part of the Wnt/Beta-catenin signaling pathway, which plays a crucial role in the development of various cancers, including gastric and brain cancers [49]. There is limited consensus in the literature regarding the role of BASP-1 in various cancers. Some studies suggest that its upregulation can impede the cell growth and metastasis of gastric tumors, while in other malignancies like gliomas, its downregulation is linked to unfavorable treatment responses and poorer patient outcomes [50,51]. Interestingly, in small-cell lung cancer (SCLC), some literature suggests that the downregulation of BASP-1 inhibits invasion and proliferation, underscoring its potential significance in SCLC development and progression [49]. On the other hand, the expression of AL1A1 was found to be increased in lung cancer induced by the tobacco-specific nitrosamine 4-(methylnitrosamino)-1-(3-pyridyl)-1-butanone (NNK) in rats [52]. This suggests that the downregulation of AL1A1 may have a role in the development of lung cancer due to the loss of detoxifying mechanisms for reactive aldehydes generated by NNK exposure [52].

Enolase is an enzyme involved in the second-to-last stage of glycolysis, where it transforms 2-phosphoglycerate into phosphoenolpyruvate. Within human cells, it appears in various forms, forming dimeric versions made up of α, β, or γ subunits. Among these variations, homodimers like αα, ββ (referred to as ENOB), and γγ are commonly found in human tissues, along with the presence of heterodimers such as αβ and αγ. Enolase 3, also known as muscle-specific enolase (MSE) or beta enolase (ENOB), is encoded by the ENO3 gene and consists of isoenzymes that specifically contain the β subunit, which is exclusively present in muscle cells. The role of ENOB in cancer is a subject of controversy, as its effects vary depending on the cell type. In some cases, ENOB has been found to be highly expressed in STK11 mutant lung cancer and colorectal cancer, associated with poor patient outcomes. Conversely, in pancreatic carcinoma and hepatocellular carcinoma (HCC), opposite trends have been observed. Possible mechanisms underlying the association between downregulation of ENOB and cancer can include the influence of ENOB on the protein level of β-catenin, while the JAK/STAT and PI3K/AKT signaling pathways remained unaffected, as found by a recent study exploring the association between downregulation of ENOB and HCC [53]. Although Nomura et al. and Fukuda et al. indicated that ENOB was downregulated in SCLC compared with LCNEC, Fukuda et al. found gamma enolase, another isoform of enolase, to be upregulated in SCLC [33,35]. Zhou et al. reported that neuron-specific enolase (NSE), closely related to ENOB and gamma enolase, is a prognostic factor in patients with SCLC [54]. The authors suggest measuring NSE levels may help predict treatment response and overall survival in SCLC patients [54]. SEGN was differentially expressed in two studies. Nakamura et al. reported downregulation of SEGN in SCLC, while Nomura et al. reported upregulation, even though both studies used FFPE tumor samples and the LC-MS/MS proteomic technique. In alignment with our observation, Baykara et al. revealed that SEGN is a potential marker for diagnosing lung neuroendocrine carcinoma (LNEC), which includes both SCLC and LCNEC [21,55]. Therefore, the authors suggested that SEGN may be a useful diagnostic tool for distinguishing LNEC from other lung cancers but not for distinguishing SCLC and LCNEC. However, validation and replication studies are needed to confirm their diagnostic utility [55]. These results demonstrate the complexity of protein expression patterns in different lung cancer subtypes and emphasize the importance of carefully considering experimental techniques when interpreting findings.

There are several limitations to the included studies. The majority of the studies were from Asian countries, and some had relatively small sample sizes. This limits the generalizability of our results. We acknowledge that the predominant focus of the reviewed literature lies in expressional studies, reflecting the available research landscape. While we have made efforts to incorporate various types of studies, including prognostic and functional, this limitation should be noted, as it may impact the extraction of robust biological and clinical correlates. In addition, the exclusion of cell line studies and animal studies and the inclusion of only English-language publications (publication bias) may represent additional limitations of the current systematic review. The exclusion of animal studies and cell lines was due to the differences in protein composition of these tissues compared with human tissues. Moreover, there is limited use of incorporating patient-matched non-tumorous tissue controls in these studies for a more precise evaluation of SCLC diagnostic biomarkers. Instead, they utilize healthy controls for comparisons. Additionally, there were a lot of discrepancies amongst the included studies, which complicated any attempts to perform a meta-analysis. Variations in sample types and preparation methods, the mass spectrometry techniques employed, and the threshold criteria for fold change analysis made it challenging to directly compare the outcomes of these investigations. Moreover, discrepancies in protein nomenclature among the included studies were also problematic and were resolved by employing UniProt’s database to ensure uniform reporting of protein names. Such differences in methodologies, analyses, and reporting techniques have been shown to account for inconsistencies among studies on the same biomarkers [56]. Hence, such studies lack sufficient information that allows for drawing meaningful conclusions on these biomarkers. This could be one of the main reasons why it was difficult to identify common markers across the different studies included. Ultimately, there is a need for a more standardized approach for conducting and reporting studies assessing biomarkers. This is through adherence to standardized reporting guidelines like the REMARK, which ensures studies publish sufficient information on how they were designed, conducted, and performed to determine whether their outcomes could have clinical utility [57].

From a clinical perspective, most of the identified markers still have limited diagnostic, prognostic, and therapeutic values. In order for these markers to be considered when making patient-specific decisions, they need to have both analytical validity and clinical utility [56,58]. The latter requires high levels of evidence that shows patients whose markers have been utilized in guiding clinical decisions have better outcomes than those whose marks have not been considered [56,59,60,61]. None of the markers identified in our study have been validated and approved in a clinical setting as a diagnostic, prognostic, or predictive biomarker. This is of critical relevance for SCLC, given that this cancer has no approved predictive biomarkers not only for conventional chemotherapy (platinum-based) but also for immunotherapy with immune checkpoint inhibitors (e.g., atezolizumab). Also, there is an unmet need to correlate the proteomic features (protein expression data) with the status of the corresponding genes (DNA and mRNA expressions).

To the best of our knowledge, this study represents the first comprehensive overview of the current state of SCLC proteomics. It emphasizes the necessity of developing a diagnostic and prognostic panel of proteins to facilitate early diagnosis and personalized treatment for the disease. However, the proteins identified through this review remain inconclusive and require further validation through larger, well-controlled studies. Furthermore, it is important to note that the reviewed studies typically employ a comprehensive analysis using MS-based proteomics encompassing all cell types present in the samples, including tumor cells, various non-neoplastic cells, and frequently abundant necrotic cells, which is characteristic of SCLC. To address these complexities and potentially enhance the specificity and sensitivity of biomarker discovery, emerging techniques such as single-cell proteomics (Sc-proteomics) hold promise for more precisely characterizing tumor cell proteomes, thus offering potential advancements in biomarker applicability. Future research should also focus on accurately identifying biomarkers that can differentiate between SCLC and LCNEC. Additionally, efforts should be made to standardize sample preparation and proteomic techniques to minimize study inconsistencies. Lastly, a multi-omics approach that combines proteomics, genomics, and metabolomics should be employed to comprehensively understand SCLC’s molecular mechanisms.

## 5. Conclusions

In conclusion, our review of 16 studies comparing protein expression in small-cell lung cancer (SCLC) with healthy controls or large-cell neuroendocrine carcinoma (LCNEC) revealed many unique proteins differentially expressed in SCLC compared to healthy control subjects or LCNEC. Five of these proteins (HPT, COTL-1, HBA, HBB, and DESP) were consistently found in at least two SCLC vs. healthy control studies, while four proteins (ENOB, BASP-1, AL1A1, and SEGN) were common in at least two SCLC vs. LCNEC studies. While our study constitutes a meaningful advance in the ongoing effort to discover effective biomarkers for SCLC diagnosis, it is crucial to note that these findings need further validation for clinical application. This entails conducting additional studies that incorporate larger and more diverse patient groups. The in-depth clinical validation and correlation of these identified biomarkers are of paramount importance, not only for enhancing diagnostic accuracy but also for informing therapeutic strategies for patients with SCLC.

## Figures and Tables

**Table 1:** Proteins differentially expressed between SCLC and healthy controls.

Author Year	Sample Size	Country	Type of Sample	Proteomic Method Utilized	Type of Analysis	Proteins Identified
Ahn et al., 2014 [20]	SCLC = 50 Controls = 29	Republic of Korea	Serum	LC/MRM-MS	Expressional	Upregulated in SCLC: SAMP, Fuscosylated-SAMP, CO9, Fucosylated-CO9, PON1, Fucosylated-PON1, Fusosylated- KAIN
Bharti et al., 2004 [21]	SCLC = 17 Controls = 5	USA	Serum	MALDI-TOF-MS	Expressional	Upregulated in SCLC: HPA, HGF
Fahrmann et al., 2021 [22]	SCLC = 15 Controls = 15	USA	Plasma	LC MS/MS	Expressional	Upregulated in SCLC: ENOG, CTRO, DIAP3, FBX11, TSP1, 1433Z, ACTB, ATCBL2, ACTC, COTL1, URP2, PROF1, TPM3, TPM4, VINC
Han et al., 2012 [23]	SCLC = 60 Controls = 48	China	Serum	SELDI-TOF MS	Expressional	Upregulated in SCLC: S10A9
Hye-Cheol et al., 2011 [24]	SCLC = 6 Controls = 6	Republic of Korea	Tumor tissue (FFPE)	MALDI-TOF MS	Expressional	Upregulated in SCLC: ACTG, TUBA1B, LAMB1, COTL1, UCH1, UBE2K, CAH11
Kang et al., 2010 [25]	SCLC = 40 Controls = 201	Republic of Korea	Blood serum	Untargeted LC-ESI-MS/MS	Expressional	Upregulated in SCLC: HPB
Lee et al., 2012 [26]	SCLC = 7 Controls = 13	Republic of Korea	Tumor tissue (FFPE)	MALDI-TOF MS	Expressional	Upregulated in SCLC: H4 Downregulated in SCLC: S10A6
Lv et al., 2020 [27]	SCLC = 72 Controls = 72	China	serum and urine samples	MALDI-TOF MS	Expressional	Upregulated in SCLC: FIBA, G6PI, CDK1
Pedersen et al., 2022 [28]	SCLC = 24 Controls = 24	Denmark	Plasma-derived microvesicles and exosomes	Nano LC-MS/MS	Expressional	Micovesicular proteins Upregulated in SCLC: SAA1, CRP, TFR1, AMPN, LG3BP Downregulated in SCLC: PGRP2, HBD, HBB, GELS, BGH3 Exosomal proteins Upregulated in SCLC: SAA1, SAA2, AMPN, HPT, FHR4 Downregulated in SCLC: KV401, FCN2, FA11, F13A, HBA
Shah et al., 2010 [29]	SCLC = 8 Controls = 8	USA	Serum	MALDI-TOF-MS, ES-MS-MS	Expressional	Upregulated in SCLC: HPT
Sugár et al., 2022 [30]	SCLC = 10 Controls = 9	Hungary	FFPE human tissue sections	nanoUHPLC-MS	Expressional Functional	Upregulated in SCLC: DESP, PSPC1, SSRP1, ACL6A, GORS2, NP1L1 Downregulated in SCLC: VWF, UTRN, EHD4, FKBP2, SUMF2, CO6A1, C4BPA, TPM2, S10A4, EIF1, CO6A2, GILT, GAPR1, ANK1, CO1A2, CATA, MEAK7, CAVN2, PDLI2, HBA, FHL1, NID1, LAMC1, HBB, CAH1, ANXA3, LYSC, AOC3, CAV1, ADH1B, CATZ, CAVN1, DESM, TENX
Zhang et al., 2021 [31]	SCLC = 9 Controls = 9	China	Urine	LC-MS/MS	Expressional	Upregulated glycosylated proteins in SCLC: CATC, MA2B2, GNS, CATD, IGHG1, PCP, IGHV3, HEXA, KV133, PLBL2 Downregulated glycosylated proteins in SCLC: DSC1, QPCT, TGC, ANXA2, DESP
Zhou et al., 2020 [32]	SCLC = 4 Controls = 3	USA	Bronchoalveolar lavage	LC-MS/MS	Expressional	Upregulated in SCLC: GNPTG, PI16, PERM, DIAC, POSTN, ITAL, PLXB2

**Table 2:** Proteins differentially expressed between SCLC and LCNEC.

Author Year	Sample Size	Country	Type of Sample	Proteomic Method Utilized	Type of Analysis	Proteins Identified
Fukuda et al., 2017 [33]	LCNEC = 10 SCLC = 10	Japan	FFPE tumor tissue	LC-MS/MS	Expressional	Upregulated in SCLC: BASP1, ENOG Downregulated in SCLC: 4F2, AL1A1, APOA1, ENOB, KCRB, LG3BP, PEBP1
Nakamura et al., 2019 [34]	SCLC = 6 LCNEC = 6	Japan	FFPE tumor tissue	LC-MS/MS	Expressional	Upregulated in SCLC: PARP9, DTX3L, HLTF, E2AK2, CNOT1, CDN2C, KAPCA, TOP1, NICA, RABP2 Downregulated in SCLC: KCD12, KCRU, SHLB2, EMAL2, CMGA, PRKRA, ERF1, HXK1, SEGN, MK01, MP2K1, RING1
Nomura et al., 2011 [35]	SCLC = 5 LCNEC = 5	Republic of Korea	FFPE tumor tissue	LC-MS/MS	Expressional	Upregulated in SCLC: BASP1, SEGN, FSCN1, NCAM1 Downregulated in SCLC: AL1A1, AK1C1, AK1C3, CD44, FABP7, ENOB

## Data Availability

The data used in this work are available upon reasonable request from the corresponding author.

## Supplementary Materials

The following supporting information can be downloaded at: https://www.mdpi.com/article/10.3390/cancers15205005/s1, Table S1: Prisma checklist; Table S2: Excluded articles at full-text screening; Table S3: Quality assessment scores.

## References

[1] Gaspar L.E., McNamara E.J., Gay E.G., Putnam J.B., Crawford J., Herbst R.S., Bonner J.A. (2012). Small-cell lung cancer: Prognostic factors and changing treatment over 15 years. Clin. Lung Cancer.

[2] Rudin C.M., Brambilla E., Faivre-Finn C., Sage J. (2021). Small-cell lung cancer. Nat. Rev. Dis. Primers.

[3] Who E.B. (2021). WHO Classification Thoracic Tumours.

[4] Szeitz B., Megyesfalvi Z., Woldmar N., Valko Z., Schwendenwein A., Barany N., Paku S., Laszlo V., Kiss H., Bugyik E. (2022). In-depth proteomic analysis reveals unique subtype-specific signatures in human small-cell lung cancer. Clin. Transl. Med..

[5] Derks J.L., Leblay N., Lantuejoul S., Dingemans A.C., Speel E.M., Fernandez-Cuesta L. (2018). New Insights into the Molecular Characteristics of Pulmonary Carcinoids and Large Cell Neuroendocrine Carcinomas, and the Impact on Their Clinical Management. J. Thorac. Oncol..

[6] Bobos M., Hytiroglou P., Kostopoulos I., Karkavelas G., Papadimitriou C.S. (2006). Immunohistochemical distinction between merkel cell carcinoma and small cell carcinoma of the lung. Am. J. Dermatopathol..

[7] Hiroshima K., Iyoda A., Shida T., Shibuya K., Iizasa T., Kishi H., Tanizawa T., Fujisawa T., Nakatani Y. (2006). Distinction of pulmonary large cell neuroendocrine carcinoma from small cell lung carcinoma: A morphological, immunohistochemical, and molecular analysis. Mod. Pathol..

[8] Kontogianni K., Nicholson A.G., Butcher D., Sheppard M.N. (2005). CD56: A useful tool for the diagnosis of small cell lung carcinomas on biopsies with extensive crush artefact. J. Clin. Pathol..

[9] Krpina K., Vranic S., Tomic K., Samarzija M., Baticic L. (2023). Small Cell Lung Carcinoma: Current Diagnosis, Biomarkers, and Treatment Options with Future Perspectives. Biomedicines.

[10] Nicholson A.G., Tsao M.S., Beasley M.B., Borczuk A.C., Brambilla E., Cooper W.A., Dacic S., Jain D., Kerr K.M., Lantuejoul S. (2022). The 2021 WHO Classification of Lung Tumors: Impact of Advances Since 2015. J. Thorac. Oncol..

[11] Travis W.D., Brambilla E., Nicholson A.G., Yatabe Y., Austin J.H.M., Beasley M.B., Chirieac L.R., Dacic S., Duhig E., Flieder D.B. (2015). The 2015 World Health Organization Classification of Lung Tumors: Impact of Genetic, Clinical and Radiologic Advances Since the 2004 Classification. J. Thorac. Oncol..

[12] George J., Walter V., Peifer M., Alexandrov L.B., Seidel D., Leenders F., Maas L., Müller C., Dahmen I., Delhomme T.M. (2018). Integrative genomic profiling of large-cell neuroendocrine carcinomas reveals distinct subtypes of high-grade neuroendocrine lung tumors. Nat. Commun..

[13] Moreira A.L., Thornton R.H. (2012). Personalized medicine for non-small-cell lung cancer: Implications of recent advances in tissue acquisition for molecular and histologic testing. Clin. Lung Cancer.

[14] Rolfo C., Castiglia M., Hong D., Alessandro R., Mertens I., Baggerman G., Zwaenepoel K., Gil-Bazo I., Passiglia F., Carreca A.P. (2014). Liquid biopsies in lung cancer: The new ambrosia of researchers. Biochim. Biophys. Acta.

[15] Villalobos-Manzo R., Rios-Castro E., Hernandez-Hernandez J.M., Oza G., Medina M.A., Tapia-Ramirez J. (2022). Identification of Transferrin Receptor 1 (TfR1) Overexpressed in Lung Cancer Cells, and Internalization of Magnetic Au-CoFe_2_O_4_ Core-Shell Nanoparticles Functionalized with Its Ligand in a Cellular Model of Small Cell Lung Cancer (SCLC). Pharmaceutics.

[16] Clark J.M., Sanders S., Carter M., Honeyman D., Cleo G., Auld Y., Booth D., Condron P., Dalais C., Bateup S. (2020). Improving the translation of search strategies using the Polyglot Search Translator: A randomized controlled trial. J. Med. Libr. Assoc..

[17] Ouzzani M., Hammady H., Fedorowicz Z., Elmagarmid A. (2016). Rayyan—A web and mobile app for systematic reviews. Syst. Rev..

[18] Lumbreras B., Porta M., Marquez S., Pollan M., Parker L.A., Hernandez-Aguado I. (2008). QUADOMICS: An adaptation of the Quality Assessment of Diagnostic Accuracy Assessment (QUADAS) for the evaluation of the methodological quality of studies on the diagnostic accuracy of ‘-omics’-based technologies. Clin. Biochem..

[19] Whiting P., Rutjes A.W., Reitsma J.B., Bossuyt P.M., Kleijnen J. (2003). The development of QUADAS: A tool for the quality assessment of studies of diagnostic accuracy included in systematic reviews. BMC Med. Res. Methodol..

[20] Ahn J.M., Sung H.J., Yoon Y.H., Kim B.G., Yang W.S., Lee C., Park H.M., Kim B.J., Lee S.Y., An H.J. (2014). Integrated Glycoproteomics Demonstrates Fucosylated Serum Paraoxonase 1 Alterations in Small Cell Lung Cancer. Mol. Cell. Proteom..

[21] Bharti A., Ma P.C., Maulik G., Singh R., Khan E., Skarin A.T., Salgia R. (2004). Haptoglobin alpha-subunit and hepatocyte growth factor can potentially serve as serum tumor biomarkers in small cell lung cancer. Anticancer Res..

[22] Fahrmann J.F., Katayama H., Irajizad E., Chakraborty A., Kato T., Mao X., Park S., Murage E., Rusling L., Yu C.Y. (2021). Plasma Based Protein Signatures Associated with Small Cell Lung Cancer. Cancers.

[23] Han M., Dai J., Zhang Y., Lin Q., Jiang M., Xu X., Liu Q., Jia J. (2012). Support vector machines coupled with proteomics approaches for detecting biomarkers predicting chemotherapy resistance in small cell lung cancer. Oncol. Rep..

[24] Hye-Cheol J., Gwang-Il K., Sang-Ho C., Kwang-Hyung L., Jung-Jae K., Jeong-Hee K., Kwang-Hoe C. (2011). Proteomic analysis of human small cell lung cancer tissues: Up-regulation of coactosin-like protein-1. J. Proteome Res..

[25] Kang S.M., Sung H.J., Ahn J.M., Park J.Y., Lee S.Y., Park C.S., Cho J.Y. (2011). The Haptoglobin β chain as a supportive biomarker for human lung cancers. Mol. Biosyst..

[26] Lee H.S., Park J.W., Chertov O., Colantonio S., Simpson J.T., Fivash M.J., Yoo C.W., Lee G.K., Zo J.I., Kim H.T. (2012). Matrix-assisted laser desorption/ionization mass spectrometry reveals decreased calcylcin expression in small cell lung cancer. Pathol. Int..

[27] Lv P., Liu Z., Xu B., Tang C., Li X., Qin H., Yang S., Gao H., He K., Liu X. (2020). Exploratory study on application of MALDI-TOF-MS to detect serum and urine peptides related to small cell lung carcinoma. Mol. Med. Rep..

[28] Pedersen S., Jensen K.P., Honore B., Kristensen S.R., Pedersen C.H., Szejniuk W.M., Maltesen R.G., Falkmer U. (2022). Circulating microvesicles and exosomes in small cell lung cancer by quantitative proteomics. Clin. Proteom..

[29] Shah A., Singh H., Sachdev V., Lee J., Yotsukura S., Salgia R., Bharti A. (2010). Differential Serum Level of Specific Haptoglobin Isoforms in Small Cell Lung Cancer. Curr. Proteom..

[30] Sugar S., Bugyi F., Toth G., Papay J., Kovalszky I., Tornoczky T., Drahos L., Turiak L. (2022). Proteomic Analysis of Lung Cancer Types-A Pilot Study. Cancers.

[31] Zhang Z.Y., Cheng X.Y., Jiang H.L., Gu J.Y., Yin Y.F., Shen Z.J., Xu C.G., Pu Z.J., Li J.B., Xu G.Q. (2021). Quantitative proteomic analysis of glycosylated proteins enriched from urine samples with magnetic ConA nanoparticles identifies potential biomarkers for small cell lung cancer. J. Pharm. Biomed. Anal..

[32] Zhou Y.Y., Yang W.M., Ao M.H., Hoti N., Gabrielson E., Chan D.W., Zhang H., Li Q.K. (2020). Proteomic Analysis of the Air-Way Fluid in Lung Cancer. Detection of Periostin in Bronchoalveolar Lavage (BAL). Front. Oncol..

[33] Fukuda T., Nomura M., Kato Y., Tojo H., Fujii K., Nagao T., Bando Y., Fehniger T.E., Marko-Varga G., Nakamura H. (2017). A selected reaction monitoring mass spectrometric assessment of biomarker candidates diagnosing large-cell neuroendocrine lung carcinoma by the scaling method using endogenous references. PLoS ONE.

[34] Nakamura H., Fujii K., Gupta V., Hata H., Koizumu H., Hoshikawa M., Naruki S., Miyata Y., Takahashi I., Miyazawa T. (2019). Identification of key modules and hub genes for small-cell lung carcinoma and large-cell neuroendocrine lung carcinoma by weighted gene co-expression network analysis of clinical tissue-proteomes. PLoS ONE.

[35] Nomura M., Fukuda T., Fujii K., Kawamura T., Tojo H., Kihara M., Bando Y., Gazdar A.F., Tsuboi M., Oshiro H. (2011). Preferential expression of potential markers for cancer stem cells in large cell neuroendocrine carcinoma of the lung. An FFPE proteomic study. J. Clin. Bioinform..

[36] Turner G.A. (1995). Haptoglobin. A potential reporter molecule for glycosylation changes in disease. Adv. Exp. Med. Biol..

[37] Thompson S., Turner G.A. (1987). Elevated levels of abnormally-fucosylated haptoglobins in cancer sera. Br. J. Cancer.

[38] Ahmed N., Barker G., Oliva K.T., Hoffmann P., Riley C., Reeve S., Smith A.I., Kemp B.E., Quinn M.A., Rice G.E. (2004). Proteomic-based identification of haptoglobin-1 precursor as a novel circulating biomarker of ovarian cancer. Br. J. Cancer.

[39] Kossowska B., Ferens-Sieczkowska M., Gancarz R., Passowicz-Muszynska E., Jankowska R. (2005). Fucosylation of serum glycoproteins in lung cancer patients. Clin. Chem. Lab. Med..

[40] Chen J., Cheuk I.W., Siu M.T., Yang W., Cheng A.S., Shin V.Y., Kwong A. (2020). Human haptoglobin contributes to breast cancer oncogenesis through glycolytic activity modulation. Am. J. Cancer Res..

[41] Provost P., Doucet J., Stock A., Gerisch G., Samuelsson B., Radmark O. (2001). Coactosin-like protein, a human F-actin-binding protein: Critical role of lysine-75. Biochem. J..

[42] Rakonjac M., Fischer L., Provost P., Werz O., Steinhilber D., Samuelsson B., Radmark O. (2006). Coactosin-like protein supports 5-lipoxygenase enzyme activity and up-regulates leukotriene A4 production. Proc. Natl. Acad. Sci. USA.

[43] Pidgeon G.P., Lysaght J., Krishnamoorthy S., Reynolds J.V., O’Byrne K., Nie D., Honn K.V. (2007). Lipoxygenase metabolism: Roles in tumor progression and survival. Cancer Metastasis Rev..

[44] Xia L., Xiao X., Liu W.L., Song Y., Liu T.J.J., Li Y.J., Zacksenhaus E., Hao X.J., Ben-David Y. (2018). Coactosin-like protein CLP/Cotl1 suppresses breast cancer growth through activation of IL-24/PERP and inhibition of non-canonical TGFβ signaling. Oncogene.

[45] Song Q.B., Hu W.G., Wang P., Yao Y., Zeng H.Z. (2011). Identification of serum biomarkers for lung cancer using magnetic bead-based SELDI-TOF-MS. Acta Pharmacol. Sin..

[46] Park J.H., Kim Y.S., Lee H.L., Shim J.Y., Lee K.S., Oh Y.J., Shin S.S., Choi Y.H., Park K.J., Park R.W. (2006). Expression of peroxiredoxin and thioredoxin in human lung cancer and paired normal lung. Respirology.

[47] Wu F., Yang S., Tang X., Liu W., Chen H., Gao H. (2020). Prognostic value of baseline hemoglobin-to-red blood cell distribution width ratio in small cell lung cancer: A retrospective analysis. Thorac. Cancer.

[48] Yang L., Chen Y., Cui T., Knosel T., Zhang Q., Albring K.F., Huber O., Petersen I. (2012). Desmoplakin acts as a tumor suppressor by inhibition of the Wnt/beta-catenin signaling pathway in human lung cancer. Carcinogenesis.

[49] Lin C.C., Huang Y.K., Cho C.F., Lin Y.S., Lo C.C., Kuo T.T., Tseng G.C., Cheng W.C., Chang W.C., Hsiao T.H. (2020). Targeting positive feedback between BASP1 and EGFR as a therapeutic strategy for lung cancer progression. Theranostics.

[50] Li L., Meng Q., Li G., Zhao L. (2020). BASP1 Suppresses Cell Growth and Metastasis through Inhibiting Wnt/*β*-Catenin Pathway in Gastric Cancer. BioMed Res. Int..

[51] Liao X., Li Z., Zheng H., Qian W., Zhang S., Chen S., Li X., Tang M., Xu Y., Yu R. (2023). Downregulation of BASP1 Promotes Temozolomide Resistance in Gliomas via Epigenetic Activation of the FBXO32/NF-κB/MGMT Axis. Mol. Cancer Res..

[52] Asad M., Wajid S., Katare D.P., Mani R.J., Jain S.K. (2019). Differential Expression of TOM34, AL1A1, PADI2 and KLRBA in NNK Induced Lung Cancer in Wistar Rats and their Implications. Curr. Cancer Drug Targets.

[53] Cui H., Guo D., Zhang X., Zhu Y., Wang Z., Jin Y., Guo W., Zhang S. (2021). ENO3 Inhibits Growth and Metastasis of Hepatocellular Carcinoma via Wnt/β-Catenin Signaling Pathway. Front. Cell Dev. Biol..

[54] Zhou M., Wang Z., Yao Y., Zhou H., Liu M., Sun J. (2017). Neuron-specific enolase and response to initial therapy are important prognostic factors in patients with small cell lung cancer. Clin. Transl. Oncol..

[55] Baykara Y., Xiao Y., Yang D., Yakirevich E., Maleki S., Garcia-Moliner M., Wang L.J., Huang C.K., Lu S. (2022). Utility of secretagogin as a marker for the diagnosis of lung neuroendocrine carcinoma. Virchows Arch..

[56] Hayes D.F., Sauerbrei W., McShane L.M. (2023). REMARK guidelines for tumour biomarker study reporting: A remarkable history. Br. J. Cancer.

[57] Harris A.L. (2005). REporting recommendations for tumour MARKer prognostic studies (REMARK). Br. J. Cancer.

[58] Hayes D.F. (2021). Defining Clinical Utility of Tumor Biomarker Tests: A Clinician’s Viewpoint. J. Clin. Oncol..

[59] Freidlin B., McShane L.M., Korn E.L. (2010). Randomized clinical trials with biomarkers: Design issues. J. Natl. Cancer Inst..

[60] Sargent D.J., Conley B.A., Allegra C., Collette L. (2005). Clinical trial designs for predictive marker validation in cancer treatment trials. J. Clin. Oncol..

[61] Simon R., Altman D.G. (1994). Statistical aspects of prognostic factor studies in oncology. Br. J. Cancer.

