# Supplementary material for: Proteomic Profiling of Small-Cell Lung Cancer: A Systematic Review
Source: Cancers (Basel). 2023 Oct 16;15(20):5005. doi: 10.3390/cancers15205005 (PMC10605593; doi:10.3390/cancers15205005)
Supplement: Supplementary file 1 [file cancers-15-05005-s001.zip › cancers-2572235-supplementary.pdf]

Supplementary Material

Proteomic Profiling of Small Cell Lung Cancer: A Systematic Review

Table of Contents

Table S1: Prisma checklist 2

Search strategy 5

Table S2: Excluded articles at full-text screening 6

Table S3: Quality assessment scores 9

References 10

Table S1: Prisma checklist

Section and Topic	Item #	Checklist item	Location where item is reported
**TITLE**			
Title	1	Identify the report as a systematic review.	1
**ABSTRACT**			
Abstract	2	See the PRISMA 2020 for Abstracts checklist.	1
**INTRODUCTION**			
Rationale	3	Describe the rationale for the review in the context of existing knowledge.	2
Objectives	4	Provide an explicit statement of the objective(s) or question(s) the review addresses.	2
**METHODS**			
Eligibility criteria	5	Specify the inclusion and exclusion criteria for the review and how studies were grouped for the syntheses.	4
Information sources	6	Specify all databases, registers, websites, organisations, reference lists and other sources searched or consulted to identify studies. Specify the date when each source was last searched or consulted.	3
Search strategy	7	Present the full search strategies for all databases, registers and websites, including any filters and limits used.	Supplementary material
Selection process	8	Specify the methods used to decide whether a study met the inclusion criteria of the review, including how many reviewers screened each record and each report retrieved, whether they worked independently, and if applicable, details of automation tools used in the process.	4
Data collection process	9	Specify the methods used to collect data from reports, including how many reviewers collected data from each report, whether they worked independently, any processes for obtaining or confirming data from study investigators, and if applicable, details of automation tools used in the process.	4
Data items	10a	List and define all outcomes for which data were sought. Specify whether all results that were compatible with each outcome domain in each study were sought (e.g. for all measures, time points, analyses), and if not, the methods used to decide which results to collect.	5
	10b	List and define all other variables for which data were sought (e.g. participant and intervention characteristics, funding sources). Describe any assumptions made about any missing or unclear information.	5
Study risk of bias assessment	11	Specify the methods used to assess risk of bias in the included studies, including details of the tool(s) used, how many reviewers assessed each study and whether they worked independently, and if applicable, details of automation tools used in the process.	5
Effect measures	12	Specify for each outcome the effect measure(s) (e.g. risk ratio, mean difference) used in the synthesis or presentation of results.	Not applicable

Reporting bias assessment	14	Describe any methods used to assess risk of bias due to missing results in a synthesis (arising from reporting biases).	Not applicable
Certainty assessment	15	Describe any methods used to assess certainty (or confidence) in the body of evidence for an outcome.	Not applicable
**RESULTS**			
Study selection	16a	Describe the results of the search and selection process, from the number of records identified in the search to the number of studies included in the review, ideally using a flow diagram.	5, figure 1
	16b	Cite studies that might appear to meet the inclusion criteria, but which were excluded, and explain why they were excluded.	Table S2
Synthesis methods	13a	Describe the processes used to decide which studies were eligible for each synthesis (e.g. tabulating the study intervention characteristics and comparing against the planned groups for each synthesis (item #5)).	6, 7
	13b	Describe any methods required to prepare the data for presentation or synthesis, such as handling of missing summary statistics, or data conversions.	6, 7
	13c	Describe any methods used to tabulate or visually display results of individual studies and syntheses.	6, 7
	13d	Describe any methods used to synthesize results and provide a rationale for the choice(s). If meta-analysis was performed, describe the model(s), method(s) to identify the presence and extent of statistical heterogeneity, and software package(s) used.	Not applicable
	13e	Describe any methods used to explore possible causes of heterogeneity among study results (e.g. subgroup analysis, meta-regression).	Not applicable
	13f	Describe any sensitivity analyses conducted to assess robustness of the synthesized results.	Not applicable
Study characteristics	17	Cite each included study and present its characteristics.	6, table 1, table 2
Risk of bias in studies	18	Present assessments of risk of bias for each included study.	Table S3
Results of individual studies	19	For all outcomes, present, for each study: (a) summary statistics for each group (where appropriate) and (b) an effect estimate and its precision (e.g. confidence/credible interval), ideally using structured tables or plots.	Table 1, table 2
Results of syntheses	20a	For each synthesis, briefly summarise the characteristics and risk of bias among contributing studies.	Table S3
	20b	Present results of all statistical syntheses conducted. If meta-analysis was done, present for each the summary estimate and its precision (e.g. confidence/credible interval) and measures of statistical heterogeneity. If comparing groups, describe the direction of the effect.	Not applicable
	20c	Present results of all investigations of possible causes of heterogeneity among study results.	Not applicable
	20d	Present results of all sensitivity analyses conducted to assess the robustness of the synthesized results.	Not applicable
Reporting biases	21	Present assessments of risk of bias due to missing results (arising from reporting biases) for each synthesis assessed.	6,7

Certainty of evidence	22	Present assessments of certainty (or confidence) in the body of evidence for each outcome assessed.	6, 7
**DISCUSSION**			
Discussion	23a	Provide a general interpretation of the results in the context of other evidence.	7, 8
	23b	Discuss any limitations of the evidence included in the review.	9, 10
	23c	Discuss any limitations of the review processes used.	10
	23d	Discuss implications of the results for practice, policy, and future research.	8,9
**OTHER INFORMATION**			
Registration and protocol	24a	Provide registration information for the review, including register name and registration number, or state that the review was not registered.	3
	24b	Indicate where the review protocol can be accessed, or state that a protocol was not prepared.	3
	24c	Describe and explain any amendments to information provided at registration or in the protocol.	3
Support	25	Describe sources of financial or non-financial support for the review, and the role of the funders or sponsors in the review.	11
Competing interests	26	Declare any competing interests of review authors.	11
Availability of data, code and other materials	27	Report which of the following are publicly available and where they can be found: template data collection forms; data extracted from included studies; data used for all analyses; analytic code; any other materials used in the review.	11

Search strategy

Final search run on 05/11/2022

PubMed: 580

((("Small Cell Lung Carcinoma"[MeSH Terms] OR "small cell lung ca*"[Title/Abstract] OR "SCLC"[Title/Abstract] OR (("Neuroendocrine Tumors"[MeSH Terms] AND "carcinoma, large cell"[MeSH Terms]) OR "large cell neuroendocrine ca*"[Title/Abstract] OR "LCNEC"[Title/Abstract]))
AND ("Proteomics"[MeSH Terms] OR "proteom*"[Title/Abstract]))
NOT "Review"[Publication Type]) NOT ("animals"[MeSH Terms] NOT "humans"[MeSH Terms])

Web of Science: 591

(((TI="small cell lung ca*" OR AB="small cell lung ca*") OR (TI=SCLC OR AB=SCLC) OR (TI="large cell neuroendocrine ca*" OR AB="large cell neuroendocrine ca*") OR (TI=LCNEC OR AB=LCNEC))

AND (TI=proteom* OR AB=proteom*)) NOT DT=(Review)

Scopus: 534

(TITLE-ABS ("small cell lung ca*") OR TITLE-ABS (sclc) OR TITLE-ABS ("large cell neuroendocrine ca*") OR TITLE-ABS (LCNEC)) AND (TITLE-ABS (proteom*)) AND (EXCLUDE (DOCTYPE , "re"))

Table S2: Excluded articles at full text screening.

Study	Title	Reason for exclusion
(An, Hong et al. 2018)	Identifying treatment options for SCLC patients with multiplexed clinical proteomic testing	Conference Abstract
(Ayyub, Saleem et al. 2016)	Glycosylated Alpha-1-acid glycoprotein 1 as a potential lung cancer serum biomarker	Wrong Outcome
(Beck, Nielsen et al. 2006)	Quantitative proteomic analysis of post-translational modifications of human histones	Non-human
(Byers, Wang et al. 2012)	Proteomic Profiling Identifies Dysregulated Pathways in Small Cell Lung Cancer and Novel Therapeutic Targets Including PARP1	Non-human
(Byers, Wang et al. 2010)	Identification of signaling pathways active in small cell lung cancer (SCLC) compared to non-small cell lung cancer (NSCLC) by proteomic profiling	Conference Abstract
(Caeser, Egger et al. 2022)	Genomic and transcriptomic analysis of a library of small cell lung cancer patient-derived xenografts	Wrong Outcome
(Campbell, Franks et al. 2018)	Chemoproteomic Discovery of a Ritanserin-Targeted Kinase Network Mediating Apoptotic Cell Death of Lung Tumor Cells	Non-human
(Cardnell, Feng et al. 2016)	Activation of the PI3K/mTOR Pathway following PARP Inhibition in Small Cell Lung Cancer	Non-human
(Cardnell and Byers 2014)	Proteomic Markers of DNA Repair and PI3K Pathway Activation Predict Response to the PARP Inhibitor BMN 673 in Small Cell Lung Cancer-Response	Letter
(Cardnell, Li et al. 2016)	Proteomic profiling identifies cMyc and TTF1 as biomarkers of response to the aurora kinase inhibitor alisertib in small cell lung cancer (SCLC)	Conference Abstract
(Cardnell, Li et al. 2017)	Protein expression of TTF1 and cMYC define distinct molecular subgroups of small cell lung cancer with unique vulnerabilities to aurora kinase inhibition, DLL3 targeting, and other targeted therapies	Wrong Outcome
(Cho, Kim et al. 2003)	Proteomic Profiling of Human Small Cell Lung Cancer Cell Line NCI-H211	Non-human
(Cho, Koh et al. 2006)	Comparative proteomics of pulmonary tumors with neuroendocrine differentiation	Wrong Outcome
(Coles, Cristea et al. 2020)	Unbiased Proteomic Profiling Uncovers a Targetable GNAS/PKA/PP2A Axis in Small Cell Lung Cancer Stem Cells	Non-human
(Dora, Rivard et al. 2020)	Neuroendocrine subtypes of small cell lung cancer differ in terms of immune microenvironment and checkpoint molecule distribution	Wrong Outcome
(Du, Yang et al. 2010)	Use of anchorchip-time-of-flight spectrometry technology to screen tumor biomarker proteins in serum for small cell lung cancer	Wrong Outcome
(Duan, Li et al. 2019)	SILAC Quantitative Proteomics and Biochemical Analyses Reveal a Novel Molecular Mechanism by Which ADAM12S Promotes the Proliferation, Migration, and Invasion of Small Cell Lung Cancer Cells through Upregulating Hexokinase 1	Non-human
(Egawa-Takata, Yoshino et al. 2018)	Small Cell Carcinomas of the Uterine Cervix and Lung: Proteomics Reveals Similar Protein Expression Profiles	Wrong Outcome
(Eriksson, Lengqvist et al. 2008)	Quantitative membrane proteomics applying narrow range peptide isoelectric focusing for studies of small cell lung cancer resistance mechanisms	Non-human
(Fujii, Miyata et al. 2018)	Differential Proteomic Analysis between Small Cell Lung Carcinoma (SCLC) and Pulmonary Carcinoid Tumors Reveals Molecular Signatures for Malignancy in Lung Cancer	Wrong Outcome
(Gao, Niu et al. 2017)	Identification of DJ-1 as a contributor to multidrug resistance in human small-cell lung cancer using proteomic analysis	Non-human
(Gong, Zhang et al. 2018)	Integrated Bioinformatics Analysis for Identificating the Therapeutic Targets of Aspirin in Small Cell Lung Cancer	Wrong Outcome

(Hassanein, Hoeksema et al. 2013)	SLC1A5 mediates glutamine transport required for lung cancer cell growth and survival	Non-human
(He, Kuhara et al. 2013)	Calretinin mediates apoptosis in small cell lung cancer cells expressing tetraspanin CD9	Non-human
(He, Naka et al. 2007)	Proteomics-based identification of α-enolase as a tumor antigen in non-small lung cancer	Wrong Outcome
(Jin, Chen et al. 2022)	Activation of PI3K/AKT Pathway Is a Potential Mechanism of Treatment Resistance in Small Cell Lung Cancer	Wrong Outcome
(Kanzaki, Ito et al. 2011)	Identification of direct targets for the miR-17-92 cluster by proteomic analysis	Non-human
(Kim, Song et al. 2016)	Differential regulation and synthetic lethality of exclusive RB1 and CDKN2A mutations in lung cancer	Wrong Outcome
(Kodama, Oshikawa et al. 2020)	A shift in glutamine nitrogen metabolism contributes to the malignant progression of cancer	Non-human
(Kojika, Hirano et al. 2007)	Validation analysis using immunohistochemistry of non-small cell lung cancer-associated proteins detected by two-dimensional polyacrylamide gel electrophoresis	Wrong Outcome
(Komtsu, Nishiyama et al. 2011)	The Proteome Analysis of Human Lung Large Cell Neuroendocrine Carcinoma	Conference Abstract
(Kundu, Cardnell et al. 2021)	SLFN11 biomarker status predicts response to lurbinectedin as a single agent and in combination with ATR inhibition in small cell lung cancer	Non-human
(Lai, Lu et al. 2006)	Secretagogin, a novel neuroendocrine marker, has a distinct expression pattern from chromogranin A	Wrong Outcome
(Lazar, Kovacs et al. 2022)	Detection of leucine-rich alpha-2-glycoprotein 1 containing immunocomplexes in the plasma of lung cancer patients with epitope-specific mAbs	Wrong Outcome
(Li, Pan et al. 2020)	A novel metastatic promoter CEMIP and its downstream molecular targets and signaling pathway of cellular migration and invasion in SCLC cells based on proteome analysis	Non-human
(Li, Zhang et al. 2018)	Quantitative proteomic analysis of mitochondrial proteins differentially expressed between small cell lung cancer cells and normal human bronchial epithelial cells	Non-human
(Llabata, Torres-Diz et al. 2021)	MAX mutant small-cell lung cancers exhibit impaired activities of MGA-dependent noncanonical polycomb repressive complex	Non-human
(Lu, Song et al. 2009)	Identification of ATP synthase beta subunit (ATPB) on the cell surface as a non-small cell lung cancer (NSCLC) associated antigen	Wrong Outcome
(Luo, Deng et al. 2005)	Comparative proteome analysis of human squamous lung carcinomas and small cell lung carcinomas	Foreign Language
(Lv, Gao et al. 2018)	Comparative proteome analysis of human squamous lung carcinomas and small cell lung carcinomas	Wrong Outcome
(Ma, Liu et al. 2011)	The effect of down regulation of calcineurin A alpha by lentiviral vector-mediated RNAi on the biological behavior of small-cell lung cancer and its bone metastasis	Non-human
(Ma, Nallasura et al. 2004)	C-Met/HGF biology and signal transduction in SCLC, especially as identified through a global proteomics phospho-antibody array based approach	Conference Abstract
(Ma, Tretiakova et al. 2007)	Downstream signalling and specific inhibition of c-MET/HGF pathway in small cell lung cancer: implications for tumour invasion	Non-human
(Ma, Xu et al. 2017)	Identification of serum proteins and multivariate models for diagnosis and therapeutic monitoring of lung cancer	Wrong Outcome
(Majumder, Hosseinian et al. 2022)	Integrated Proteomics-Based Physical and Functional Mapping of AXL Kinase Signaling Pathways and Inhibitors Define Its Role in Cell Migration	Non-human
(Mao, Zheng et al. 2021)	Exosomal miR-375-3p breaks vascular barrier and promotes small cell lung cancer metastasis by targeting claudin-1	Wrong Outcome

(Masalu, Hosea et al. 2014)	Glucose regulated protein 78 (GRP 78) as a cytoprotection against apoptosis in Small Cell Lung Carcinoma	Non-human
(McKitterick, Bicak et al. 2020)	Facilitating serum determination of neuron specific enolase at clinically relevant levels by coupling on-line molecularly imprinted solid-phase extraction to LC-MS/MS	Wrong Outcome
(McKitterick, Bicak et al. 2021)	On-line duplex molecularly imprinted solid-phase extraction for analysis of low-abundant biomarkers in human serum by liquid chromatography-tandem mass spectrometry	Wrong Outcome
(Megyesfalvi, Barany et al. 2022)	Expression patterns and prognostic relevance of subtype-specific transcription factors in surgically resected small-cell lung cancer: an international multicenter study	Non-human
(Miyagawa-Hayashino, Okada et al. 2021)	TTF-1 and c-MYC-defined Phenotypes of Large Cell Neuroendocrine Carcinoma and Delta-like Protein 3 Expression for Treatment Selection	Wrong Outcome
(Nagashio, Sato et al. 2010)	Significant high expression of cytokeratins 7, 8, 18, 19 in pulmonary large cell neuroendocrine carcinomas, compared to small cell lung carcinomas	Non-human
(Nasgashio, Sato et al. 2011)	The balance between the expressions of hASH1 and HES1 differs between large cell neuroendocrine carcinoma and small cell carcinoma of the lung	Wrong Outcome
(Nguyen, Markaki et al. 2019)	Proteomics analysis discovers biomarkers in serum months to years before small cell lung cancer: The HUNT study	Conference Abstract
(Nishimura, Kawamura et al. 2014)	Clinical initiatives linking Japanese and Swedish healthcare resources on cancer studies utilizing Biobank Repositories	Wrong Outcome
(Nomura, Nishimura et al. 2010)	Proteomic Analysis Of Large Cell Neuroendocrine Carcinoma, Small Cell Lung Carcinoma And Large Cell Carcinoma From Formalin-Fixed Paraffin Embedded Samples And Validation By Immunehistochemical Staining	Conference Abstract
(Nomura, Nishimura et al. 2009)	A cell line from large cell neuroendocrine carcinoma (LCNEC) of the lung and its genetic and proteomic analysis	Conference Abstract
(Palve, Knezevic et al. 2022)	The non-canonical target PARP16 contributes to polypharmacology of the PARP inhibitor talazoparib and its synergy with WEE1 inhibitors	Non-human
(Perzanowska, Fatalska et al. 2018)	An MRM-Based Cytokeratin Marker Assay as a Tool for Cancer Studies: Application to Lung Cancer Pleural Effusions	Wrong Outcome
(Planque, Kulasingam et al. 2009)	Identification of Five Candidate Lung Cancer Biomarkers by Proteomics Analysis of Conditioned Media of Four Lung Cancer Cell Lines	Non-human
(Poschmann, Lendzian et al. 2013)	A combination of two electrophoretical approaches for detailed proteome-based characterization of SCLC subtypes	Non-human
(Prabhu, Scott et al. 2020)	Integrated Proteometabolomic Analysis Reveals Metabolic Vulnerabilities in Small-Cell Lung Cancer	Conference Abstract
(Rajasekaran, Siddiqui et al. 2021)	Integrated multi-omics analysis of RB-loss identifies widespread cellular programming and synthetic weaknesses	Wrong Outcome
(Rikova, Hall et al. 2016)	Proteomic analysis identifies multi-dimensional deregulated signaling pathways in SCLC lung cancer	Conference Abstract
(Rosell and Wannesson 2012)	A genetic snapshot of small cell lung cancer	Non-human
(Seike, Kondo et al. 2005)	Proteomic signatures for histological types of lung cancer	Wrong Outcome
(Sen, Tong et al. 2017)	Targeting AXL and mTOR Pathway Overcomes Primary and Acquired Resistance to WEE1 Inhibition in Small-Cell Lung Cancer	Non-human
(Sen, Tong et al. 2017)	CHK1 Inhibition in Small-Cell Lung Cancer Produces Single-Agent Activity in Biomarker-Defined Disease Subsets and Combination Activity with Cisplatin or Olaparib	Non-human
(Sen, Tong et al. 2016)	Proteomic profiling identifies ATM expression level as a predictive biomarker to ATR and PARP inhibition in small cell lung cancer (SCLC) Authors: Sen, T.; Tong	Non-human

(Stewart, Tong et al. 2017)	Dynamic variations in epithelial-to-mesenchymal transition (EMT), ATM, and SLFN11 govern response to PARP inhibitors and cisplatin in small cell lung cancer	Non-human
(Stewart, Li et al. 2015)	Integrating proteomics and metabolomics characterizes active pathways and potential drug targets in small cell lung cancer	Conference Abstract
(Sumi, Ctortecka et al. 2019)	Divergent Polypharmacology-Driven Cellular Activity of Structurally Similar Multi-Kinase Inhibitors through Cumulative Effects on Individual Targets	Wrong Outcome
(Szeitz, Megyesfalvi et al. 2022)	In-depth proteomic analysis reveals unique subtype-specific signatures in human small-cell lung cancer	Non-human
(Tanca, Addis et al. 2011)	Proteomic analysis of formalin-fixed, paraffin-embedded lung neuroendocrine tumor samples from hospital archives	Wrong Outcome
(Togayachi, Iwaki et al. 2017)	Glycobiomarker, Fucosylated Short-Form Secretogranin III Levels Are Increased in Serum of Patients with Small Cell Lung Carcinoma	Wrong Outcome
(Toyokawa, Kodama et al. 2021)	Comprehensive analysis of the metabolic enzymes in patients with small cell lung cancer using a large-scale targeted proteomics assay	Non-human
(Tripathi, Fahrmann et al. 2017)	MCAM Mediates Chemoresistance in Small-Cell Lung Cancer via the PI3K/AKT/SOX2 Signaling Pathway	Non-human
(Udyavar, Hoeksema et al. 2013)	Co-expression network analysis identifies Spleen Tyrosine Kinase (SYK) as a candidate oncogenic driver in a subset of small-cell lung cancer	Wrong Outcome
(Villalobos-Manzo, Ríos-Castro et al. 2022)	Identification of Transferrin Receptor 1 (TfR1) Overexpressed in Lung Cancer Cells, and Internalization of Magnetic Au-CoFe(2)O(4) Core-Shell Nanoparticles Functionalized with Its Ligand in a Cellular Model of Small Cell Lung Cancer (SCLC)	Non-human
(Wang, Zhang et al. 2015)	Association of chromosome 19 to lung cancer genotypes and phenotypes	Review
(Wu, Hao et al. 2020)	Comparative proteogenomics profiling of non-small and small lung carcinoma cell lines using mass spectrometry	Non-human
(Xiao, Zhang et al. 2012)	Proteomic analysis of human saliva from lung cancer patients using two-dimensional difference gel electrophoresis and mass spectrometry	Wrong Outcome
(Yang, Tian et al. 2016)	Serum protein profiles of patients with lung cancer of different histological types	Wrong Outcome
(Yang, Xiao et al. 2005)	Application of serum SELDI proteomic patterns in diagnosis of lung cancer	Wrong Outcome
(Yang, Zhang et al. 2006)	Analysis of differential expression proteins of small cell lung cancer and matched normal lung tissues by 2-DE and MALDI-TOF-MS	Conference Abstract
(Yin, Shi et al. 2021)	Combinations of proteasome inhibitors with obatoclax are effective for small cell lung cancer	Non-human
(Yoshimura, Takeda et al. 2021)	Discovery of Novel Biomarkers of Small Cell Lung Cancer by Proteomics of Exosomes	Conference
(Yu, Li et al. 2018)	Integrated omics and gene expression analysis identifies the loss of metabolite-metabolite correlations in small cell lung cancer	Non-human
(Yurdakul, Sonmez et al. 2012)	Evaluation of Relationship Between Serum Protein Profiles and Lung Cancer by SELDI-TOF-MS (Surface Enhanced Laser Desabsorbtion Ionization Time of Flight Mass Spectrometry) Method	Wrong Outcome
(Zhou, Lih et al. 2020)	Proteomic signatures of 16 major types of human cancer reveal universal and cancer-type-specific proteins for the identification of potential therapeutic targets	Wrong Outcome
(Ziv, Barnea et al. 2006)	Comparative proteomics of small cell lung carcinoma	Non-human

Table S3: Quality assessment scores

Study	S1	S2	S3	S4	S5	S6	S7	S8	S9	S10	S11	S12	S13	S14	S15	S16	Total
Ahn, J. M. et al.	1	0	1	1	0	0	1	1	0	1	1	0	0	0	0	0	7
Bharti, A. et al.	1	0	1	1	1	0	1	0	0	1	1	1	1	0	0	0	9
Fahrmann, J. F et al.	1	1	1	1	1	0	0	0	1	1	1	0	0	1	1	1	11
Han, M et al.	1	1	1	1	0	0	1	1	0	1	1	0	0	1	0	0	9
Hye-Cheol, J et al.	1	0	0	1	0	0	1	0	0	1	1	1	1	0	0	0	7
Kang, S et al.	0	0	0	1	0	1	0	0	1	1	0	0	0	0	1	0	5
Lee et al.	1	1	1	1	1	0	1	1	0	1	1	1	1	1	1	1	14
Lv et al.	1	1	1	1	1	0	0	0	1	1	1	0	0	1	1	1	11
Pederson et al.	1	0	1	1	1	0	1	1	0	1	1	1	1	0	0	0	10
Shah et al.	0	0	1	0	1	0	0	1	0	1	0	0	0	0	0	0	4
Sugár et al.	1	1	1	1	1	0	1	1	1	1	0	0	0	1	0	0	10
Zhang et al.	1	0	1	0	1	0	1	1	0	1	1	1	0	0	0	0	8
Zhou et al.	1	1	1	0	1	0	0	1	0	1	1	0	0	0	0	0	7
Fukuda, T et al.	0	0	1	0	0	0	1	1	1	1	0	0	0	0	0	0	5
Nakamura et al.	1	0	1	1	1	0	1	1	0	1	0	0	0	0	0	0	7
Nomura et al.	1	1	1	0	1	0	0	0	0	1	0	0	0	0	0	0	5

Reference list

An, E., S. H. Hong, H. J. An, S. P. Thyparambil, S. Sellappan, D. Y. Yan, Y. Tian, F. Cecchi, T. A. Hembrough and T. J. Kim (2018). "Identifying treatment options for SCLC patients with multiplexed clinical proteomic testing." JOURNAL OF CLINICAL ONCOLOGY **36**(15).

Ayyub, A., M. Saleem, I. Fatima, A. Tariq, N. Hashmi and S. G. Musharraf (2016). "Glycosylated Alpha-1-acid glycoprotein 1 as a potential lung cancer serum biomarker." INTERNATIONAL JOURNAL OF BIOCHEMISTRY & CELL BIOLOGY **70**: 68-75.

Beck, H. C., E. C. Nielsen, R. Matthiesen, L. H. Jensen, M. Sehested, P. Finn, M. Grauslund, A. M. Hansen and O. N. Jensen (2006). "Quantitative proteomic analysis of post-translational modifications of human histones." Mol Cell Proteomics **5**(7): 1314-1325.

Byers, L. A., J. Wang, M. B. Nilsson, J. Fujimoto, P. Saintigny, J. Yordy, U. Giri, M. Peyton, Y. H. Fan, L. X. Diao, F. Masrorpour, L. Shen, W. B. Liu, B. Duchemann, P. Tumula, V. Bhardwaj, J. Welsh, S. Weber, B. S. Glisson, N. Kalhor, Wistuba, II, L. Girard, S. M. Lippman, G. B. Mills, K. R. Coombes, J. N. Weinstein, J. D. Minna and J. V. Heymach (2012). "Proteomic Profiling Identifies Dysregulated Pathways in Small Cell Lung Cancer and Novel Therapeutic Targets Including PARP1." CANCER DISCOVERY **2**(9): 798-811.

Byers, L. A., J. Wang, J. Yordy, Y. H. Fan, U. Giri, L. Shen, I. Wistuba, L. Girard, K. Coombes, J. Weinstein, J. Minna and J. Heymach (2010). "Identification of signaling pathways active in small cell lung cancer (SCLC) compared to non-small cell lung cancer (NSCLC) by proteomic profiling." CANCER RESEARCH **70**.

Caeser, R., J. V. Egger, S. Chavan, N. D. Socci, C. B. Jones, F. E. Kombak, M. Asher, M. H. Roehrl, N. S. Shah, V. Allaj, P. Manoj, S. E. Tischfield, A. Kulick, M. Meneses, C. A. Iacobuzio-Donahue, W. V. Lai, U. Bhanot, M. K. Baine, N. Rekhtman, T. J. Hollmann, E. de Stanchina, J. T. Poirier, C. M. Rudin and T. Sen (2022). "Genomic and transcriptomic analysis of a library of small cell lung cancer patient-derived xenografts." NATURE COMMUNICATIONS **13**(1).

Campbell, S. T., C. E. Franks, A. L. Borne, M. Shin, L. Z. Zhang and K. L. Hsu (2018). "Chemoproteomic Discovery of a Ritanserin-Targeted Kinase Network Mediating Apoptotic Cell Death of Lung Tumor Cells." MOLECULAR PHARMACOLOGY **94**(5): 1246-1255.

Cardnell, R. J., Y. Feng, S. Mukherjee, L. Diao, P. Tong, C. A. Stewart, F. Masrorpour, Y. Fan, M. Nilsson, Y. Shen, J. V. Heymach, J. Wang and L. A. Byers (2016). "Activation of the PI3K/mTOR Pathway following PARP Inhibition in Small Cell Lung Cancer." PLoS One **11**(4): e0152584.

Cardnell, R. J., L. Li, T. Sen, R. Bara, P. Tong, J. Fujimoto, A. S. Ireland, M. R. Guthrie, S. Bheddah, U. Banerjee, N. N. Kalu, Y. H. Fan, S. J. Dylla, F. M. Johnson, Wistuba, II, T. G. Oliver, J. V. Heymach, B. S. Glisson, J. Wang and L. A. Byers (2017). "Protein expression of TTF1 and cMYC define distinct molecular subgroups of small cell lung cancer with unique vulnerabilities to aurora kinase inhibition, DLL3 targeting, and other targeted therapies." Oncotarget **8**(43): 73419-73432.

Cardnell, R. J., L. R. Li, F. Masrorpour, H. F. Niu, J. Ecsedy, J. Wang and L. A. Byers (2016). "Proteomic profiling identifies cMyc and TTF1 as biomarkers of response to the aurora kinase inhibitor alisertib in small cell lung cancer (SCLC)." CANCER RESEARCH **76**.

Cardnell, R. J. G. and L. A. Byers (2014). "Proteomic Markers of DNA Repair and PI3K Pathway Activation Predict Response to the PARP Inhibitor BMN 673 in Small Cell Lung Cancer-Response." CLINICAL CANCER RESEARCH **20**(8): 2237-2237.

Cho, H. Y., M. K. Kim, Y. D. Yoo, M. J. Ahn and J. S. Jang (2003). "Proteomic Profiling of Human Small Cell Lung Cancer Cell Line NCI-H211." Cancer Res Treat **35**(6): 489-496.

Cho, N. H., E. S. Koh, D. W. Lee, H. Kim, Y. P. Choi, S. H. Cho and D. S. Kim (2006). "Comparative proteomics of pulmonary tumors with neuroendocrine differentiation." JOURNAL OF PROTEOME RESEARCH **5**(3): 643-650.

Coles, G. L., S. Cristea, J. T. Webber, R. S. Levin, S. M. Moss, A. He, J. Sangodkar, Y. C. Hwang, J. Arand, A. P. Drainas, N. A. Mooney, J. Demeter, J. N. Spradlin, B. Mauch, V. Le, Y. T. Shue, J. H. Ko, M. C. Lee, C.

Kong, D. K. Nomura, M. Ohlmeyer, D. L. Swaney, N. J. Krogan, P. K. Jackson, G. Narla, J. D. Gordan, K. M. Shokat and J. Sage (2020). "Unbiased Proteomic Profiling Uncovers a Targetable GNAS/PKA/PP2A Axis in Small Cell Lung Cancer Stem Cells." Cancer Cell **38**(1): 129-143.e127.
Dora, D., C. Rivard, H. Yu, P. Bunn, K. Suda, S. Ren, S. Lueke Pickard, V. Laszlo, T. Harko, Z. Megyesfalvi, J. Moldvay, F. R. Hirsch, B. Dome and Z. Lohinai (2020). "Neuroendocrine subtypes of small cell lung cancer differ in terms of immune microenvironment and checkpoint molecule distribution." Mol Oncol **14**(9): 1947-1965.
Du, J., S. Yang, X. Lin, L. Bu, Y. Nan, S. Huo and W. Shang (2010). "Use of anchorchip-time-of-flight spectrometry technology to screen tumor biomarker proteins in serum for small cell lung cancer." Diagn Pathol **5**: 60.
Duan, Q., D. Li, L. Xiong, Z. Chang and G. Xu (2019). "SILAC Quantitative Proteomics and Biochemical Analyses Reveal a Novel Molecular Mechanism by Which ADAM12S Promotes the Proliferation, Migration, and Invasion of Small Cell Lung Cancer Cells through Upregulating Hexokinase 1." J Proteome Res **18**(7): 2903-2914.
Egawa-Takata, T., K. Yoshino, K. Hiramatsu, S. Nakagawa, S. Serada, A. Nakajima, H. Endo, S. Kubota, S. Matsuzaki, E. Kobayashi, Y. Ueda, E. Morii, M. Inoue, T. Naka and T. Kimura (2018). "Small Cell Carcinomas of the Uterine Cervix and Lung: Proteomics Reveals Similar Protein Expression Profiles." INTERNATIONAL JOURNAL OF GYNECOLOGICAL CANCER **28**(9): 1751-1757.
Eriksson, H., J. Lengqvist, J. Hedlund, K. Uhlén, L. M. Orre, B. Bjellqvist, B. Persson, J. Lehtiö and P. J. Jakobsson (2008). "Quantitative membrane proteomics applying narrow range peptide isoelectric focusing for studies of small cell lung cancer resistance mechanisms." Proteomics **8**(15): 3008-3018.
Fujii, K., Y. Miyata, I. Takahashi, H. Koizumi, H. Saji, M. Hoshikawa, M. Takagi, T. Nishimura and H. Nakamura (2018). "Differential Proteomic Analysis between Small Cell Lung Carcinoma (SCLC) and Pulmonary Carcinoid Tumors Reveals Molecular Signatures for Malignancy in Lung Cancer." PROTEOMICS CLINICAL APPLICATIONS **12**(6).
Gao, H., Y. Niu, M. Li, S. Fang and L. Guo (2017). "Identification of DJ-1 as a contributor to multidrug resistance in human small-cell lung cancer using proteomic analysis." Int J Exp Pathol **98**(2): 67-74.
Gong, L., D. Zhang, Y. Dong, Y. Lei, Y. Qian, X. Tan, S. Han and J. Wang (2018). "Integrated Bioinformatics Analysis for Identificating the Therapeutic Targets of Aspirin in Small Cell Lung Cancer." J Biomed Inform **88**: 20-28.
Hassanein, M., M. D. Hoeksema, M. Shiota, J. Qian, B. K. Harris, H. Chen, J. E. Clark, W. E. Alborn, R. Eisenberg and P. P. Massion (2013). "SLC1A5 mediates glutamine transport required for lung cancer cell growth and survival." Clin Cancer Res **19**(3): 560-570.
He, P., H. Kuhara, I. Tachibana, Y. J. Jin, Y. Takeda, S. Tetsumoto, T. Minami, S. Kohmo, H. Hirata, R. Takahashi, K. Inoue, I. Nagatomo, H. Kida, T. Kijima, T. Naka, E. Morii, I. Kawase and A. Kumanogoh (2013). "Calretinin mediates apoptosis in small cell lung cancer cells expressing tetraspanin CD9." FEBS OPEN BIO **3**: 225-230.
He, P., T. Naka, S. Serada, M. Fujimoto, T. Tanaka, S. Hashimoto, Y. Shima, T. Yamadori, H. Suzuki, T. Hirashima, K. Matsui, H. Shiono, M. Okumura, T. Nishida, I. Tachibana, N. Norioka, S. Norioka and I. Kawase (2007). "Proteomics-based identification of α-enolase as a tumor antigen in non-small lung cancer." Cancer Science **98**(8): 1234-1240.
Jin, Y., Y. Chen, H. Tang, X. Hu, S. M. Hubert, Q. Li, D. Su, H. Xu, Y. Fan, X. Yu, Q. Chen, J. Liu, W. Hong, Y. Xu, H. Deng, D. Zhu, P. Li, Y. Gong, X. Xia, C. M. Gay, J. Zhang and M. Chen (2022). "Activation of PI3K/AKT Pathway Is a Potential Mechanism of Treatment Resistance in Small Cell Lung Cancer." Clin Cancer Res **28**(3): 526-539.
Kanzaki, H., S. Ito, H. Hanafusa, Y. Jitsumori, S. Tamaru, K. Shimizu and M. Ouchida (2011). "Identification of direct targets for the miR-17-92 cluster by proteomic analysis." PROTEOMICS **11**(17): 3531-3539.

Kim, N., M. Song, S. Kim, Y. Seo, Y. Kim and S. Yoon (2016). "Differential regulation and synthetic lethality of exclusive RB1 and CDKN2A mutations in lung cancer." INTERNATIONAL JOURNAL OF ONCOLOGY **48**(1): 367-375.
Kodama, M., K. Oshikawa, H. Shimizu, S. Yoshioka, M. Takahashi, Y. Izumi, T. Bamba, C. Tateishi, T. Tomonaga, M. Matsumoto and K. I. Nakayama (2020). "A shift in glutamine nitrogen metabolism contributes to the malignant progression of cancer." NATURE COMMUNICATIONS **11**(1).
Kojika, M., T. Hirano, J. Matsubayashi, R. Guo, Y. Gong, T. Kawamura, H. Kataba, T. Ohira, K. Mukai and H. Kato (2007). "Validation analysis using immunohistochemistry of non-small cell lung cancer-associated proteins detected by two-dimensional polyacrylamide gel electrophoresis." Japanese Journal of Lung Cancer **47**(7): 861-869.
Komtsu, H., N. Nishiyama, K. Nagano, N. Izumi, K. Tei, S. Hanada, H. Wanibuchi and S. Suehiro (2011). "THE PROTEOME ANALYSIS OF HUMAN LUNG LARGE CELL NEUROENDOCRINE CARCINOMA." JOURNAL OF THORACIC ONCOLOGY **6**(6): S714-S714.
Kundu, K., R. J. Cardnell, B. N. Zhang, L. Shen, C. A. Stewart, K. Ramkumar, K. R. Cargill, J. Wang, C. M. Gay and L. A. Byers (2021). "SLFN11 biomarker status predicts response to lurbinectedin as a single agent and in combination with ATR inhibition in small cell lung cancer." TRANSLATIONAL LUNG CANCER RESEARCH **10**(11): 4095-+.
Lai, M. D., B. J. Lu, X. M. Xing, E. P. Xu, G. P. Ren and Q. Huang (2006). "Secretagogin, a novel neuroendocrine marker, has a distinct expression pattern from chromogranin A." VIRCHOWS ARCHIV **449**(4): 402-409.
Lazar, J., A. Kovacs, I. Tornyi, L. Takacs and I. Kurucz (2022). "Detection of leucine-rich alpha-2-glycoprotein 1 containing immunocomplexes in the plasma of lung cancer patients with epitope-specific mAbs." CANCER BIOMARKERS **34**(1): 113-122.
Li, L., Y. Pan, X. Mo, T. Wei, J. Song, M. Luo, G. Huang, C. Teng, K. Liang, N. Mao and J. Yang (2020). "A novel metastatic promoter CEMIP and its downstream molecular targets and signaling pathway of cellular migration and invasion in SCLC cells based on proteome analysis." J Cancer Res Clin Oncol **146**(10): 2519-2534.
Li, W., W. Zhang, W. J. Deng, Y. J. Zhong, Y. H. Zhang, Z. Peng, H. J. Chen, R. Y. Sun, X. M. Zhang and S. Y. Yang (2018). "Quantitative proteomic analysis of mitochondrial proteins differentially expressed between small cell lung cancer cells and normal human bronchial epithelial cells." THORACIC CANCER **9**(11): 1366-1375.
Llabata, P., M. Torres-Diz, A. Gomez, L. Tomas-Daza, O. A. Romero, J. Grego-Bessa, P. Llinas-Arias, A. Valencia, M. Esteller, B. M. Javierre, X. Y. Zhang and M. Sanchez-Cespedes (2021). "MAX mutant small-cell lung cancers exhibit impaired activities of MGA-dependent noncanonical polycomb repressive complex." PROCEEDINGS OF THE NATIONAL ACADEMY OF SCIENCES OF THE UNITED STATES OF AMERICA **118**(37).
Lu, Z. J., Q. F. Song, S. S. Jiang, Q. Song, W. Wang, G. H. Zhang, B. Kan, L. J. Chen, J. L. Yang, F. Luo, Z. Y. Qian, Y. Q. Wei and L. T. Gou (2009). "Identification of ATP synthase beta subunit (ATPB) on the cell surface as a non-small cell lung cancer (NSCLC) associated antigen." BMC Cancer **9**: 16.
Luo, G. A., B. Deng, N. S. Ye and Y. M. Wang (2005). "Comparative proteome analysis of human squamous lung carcinomas and small cell lung carcinomas." CHEMICAL JOURNAL OF CHINESE UNIVERSITIES-CHINESE **26**(9): 1645-1649.
Lv, J., D. Gao, Y. Zhang, D. Wu, L. Shen and X. Wang (2018). "Heterogeneity of lipidomic profiles among lung cancer subtypes of patients." J Cell Mol Med **22**(10): 5155-5159.
Ma, N. Q., L. L. Liu, J. Min, J. W. Wang, W. F. Jiang, Y. Liu, Y. G. Feng, H. C. Su, Y. M. Feng and H. L. Zhang (2011). "The effect of down regulation of calcineurin A alpha by lentiviral vector-mediated RNAi on the biological behavior of small-cell lung cancer and its bone metastasis." CLINICAL & EXPERIMENTAL METASTASIS **28**(8): 765-778.

Ma, P. C., V. Nallasura, R. Uppalapati, D. Nguyen and R. Salgia (2004). "C-Met/HGF biology and signal transduction in SCLC, especially as identified through a global proteomics phospho-antibody array based approach." LUNG CANCER **46**: S49-S50.

Ma, P. C., M. S. Tretiakova, V. Nallasura, R. Jagadeeswaran, A. N. Husain and R. Salgia (2007). "Downstream signalling and specific inhibition of c-MET/HGF pathway in small cell lung cancer: implications for tumour invasion." BRITISH JOURNAL OF CANCER **97**(3): 368-377.

Ma, R., H. Xu, J. Wu, A. Sharma, S. Bai, B. Dun, C. Jing, H. Cao, Z. Wang, J. X. She and J. Feng (2017). "Identification of serum proteins and multivariate models for diagnosis and therapeutic monitoring of lung cancer." Oncotarget **8**(12): 18901-18913.

Majumder, A., S. Hosseinian, M. Stroud, E. Adhikari, J. J. Saller, M. A. Smith, G. L. Zhang, S. Agarwal, M. Creixell, B. S. Meyer, F. Kinose, K. Bowers, B. Fang, P. A. Stewart, E. A. Welsh, T. A. Boyle, A. S. Meyer, J. M. Koomen and E. B. Haura (2022). "Integrated Proteomics-Based Physical and Functional Mapping of AXL Kinase Signaling Pathways and Inhibitors Define Its Role in Cell Migration." MOLECULAR CANCER RESEARCH **20**(4): 542-555.

Mao, S. S., S. F. Zheng, Z. L. Lu, X. F. Wang, Y. Wang, G. C. Zhang, H. Y. Xu, J. B. Huang, Y. Y. Lei, C. M. Liu, N. Sun and J. He (2021). "Exosomal miR-375-3p breaks vascular barrier and promotes small cell lung cancer metastasis by targeting claudin-1." TRANSLATIONAL LUNG CANCER RESEARCH **10**(7): 3155-+.

Masalu, R., K. Hosea, M. Meyer, S. L. Lyantagaye and B. Ndimba (2014). "Glucose regulated protein 78 (GRP 78) as a cytoprotection against apoptosis in Small Cell Lung Carcinoma." Current Trends in Biotechnology and Pharmacy **8**(2): 185-191.

McKitterick, N., T. C. Bicak, P. A. G. Cormack, L. Reubsaet and T. G. Halvorsen (2020). "Facilitating serum determination of neuron specific enolase at clinically relevant levels by coupling on-line molecularly imprinted solid-phase extraction to LC-MS/MS." Anal Chim Acta **1140**: 210-218.

McKitterick, N., T. C. Bicak, M. A. Switnicka-Plak, P. A. G. Cormack, L. Reubsaet and T. G. Halvorsen (2021). "On-line duplex molecularly imprinted solid-phase extraction for analysis of low-abundant biomarkers in human serum by liquid chromatography-tandem mass spectrometry." J Chromatogr A **1655**: 462490.

Megyesfalvi, Z., N. Barany, A. Lantos, Z. Valko, O. Pipek, C. Lang, A. Schwendenwein, F. Oberndorfer, S. Paku, B. Ferencz, K. Dezso, J. Fillinger, Z. Lohinai, J. Moldvay, G. Galffy, B. Szeitz, M. Rezeli, C. Rivard, F. R. Hirsch, L. Brcic, H. Popper, I. Kern, M. Kovacevic, J. Skarda, M. Mittak, G. Marko-Varga, K. Bogos, F. Renyi-Vamos, M. A. Hoda, T. Klikovits, K. Hoetzenecker, K. Schelch, V. Laszlo and B. Dome (2022). "Expression patterns and prognostic relevance of subtype-specific transcription factors in surgically resected small-cell lung cancer: an international multicenter study." J Pathol **257**(5): 674-686.

Miyagawa-Hayashino, A., S. Okada, N. Takeda-Miyata, Y. Takashima, T. Yamada, Y. Takemura, J. Uchino, M. Inoue, K. Takayama and E. Konishi (2021). "TTF-1 and c-MYC-defined Phenotypes of Large Cell Neuroendocrine Carcinoma and Delta-like Protein 3 Expression for Treatment Selection." APPLIED IMMUNOHISTOCHEMISTRY & MOLECULAR MORPHOLOGY **29**(4): 313-320.

Nagashio, R., Y. Sato, T. Matsumoto, T. Kageyama, Y. Satoh, S. Ryuge, N. Masuda, S. X. Jiang and I. Okayasu (2010). "Significant high expression of cytokeratins 7, 8, 18, 19 in pulmonary large cell neuroendocrine carcinomas, compared to small cell lung carcinomas." PATHOLOGY INTERNATIONAL **60**(2): 71-77.

Nasgashio, R., Y. Sato, T. Matsumoto, T. Kageyama, M. Hattori, A. Iyoda, Y. Satoh, S. Ryuge, N. Masuda, S. X. Jiang and M. Saegusa (2011). "The balance between the expressions of hASH1 and HES1 differs between large cell neuroendocrine carcinoma and small cell carcinoma of the lung." Lung Cancer **74**(3): 405-410.

Nguyen, O. T. D., M. Markaki, C. Chatzipantsiou, A. Sharma, V. Lagani, I. Tsamardinos and O. D. Roe (2019). "Proteomics analysis discovers biomarkers in serum months to years before small cell lung cancer: The HUNT study." JOURNAL OF CLINICAL ONCOLOGY **37**(15).

Nishimura, T., T. Kawamura, Y. Sugihara, Y. Bando, S. Sakamoto, M. Nomura, N. Ikeda, T. Ohira, J. Fujimoto, H. Tojo, T. Hamakubo, T. Kodama, R. Andersson, T. E. Fehniger, H. Kato and G. Marko-Varga (2014). "Clinical initiatives linking Japanese and Swedish healthcare resources on cancer studies utilizing Biobank Repositories." Clin Transl Med **3**(1): 61.
Nomura, M., T. Nishimura, T. Fukuda, K. Fujii, T. Kawamura, M. Tsuboi, N. Saijo, H. Kato, T. Ohira and N. Ikeda (2010). "PROTEOMIC ANALYSIS OF LARGE CELL NEUROENDOCRINE CARCINOMA, SMALL CELL LUNG CARCINOMA AND LARGE CELL CARCINOMA FROM FORMALIN-FIXED PARAFFIN EMBEDDED SAMPLES AND VALIDATION BY IMMUNEHISTOCHEMICAL STAINING." ANNALS OF ONCOLOGY **21**: 73-73.
Nomura, M., T. Nishimura, T. Ohira, H. Tojo, S. Nonomura, H. Endo, Y. Bando, K. Fujii, N. Ikeda and A. Gazdar (2009). "A cell line from large cell neuroendocrine carcinoma (LCNEC) of the lung and its genetic and proteomic analysis." CANCER RESEARCH **69**.
Palve, V., C. E. Knezevic, D. S. Bejan, Y. T. Luo, X. L. Li, S. Novakova, E. A. Welsh, B. Fang, F. Kinose, E. B. Haura, A. N. Monteiro, J. M. Koomen, M. S. Cohen, H. R. Lawrence and U. Rix (2022). "The non-canonical target PARP16 contributes to polypharmacology of the PARP inhibitor talazoparib and its synergy with WEE1 inhibitors." CELL CHEMICAL BIOLOGY **29**(2): 202-+.
Perzanowska, A., A. Fatalska, G. Wojtas, A. Lewandowicz, A. Michalak, G. Krasowski, C. H. Borchers, M. Dadlez and D. Domanski (2018). "An MRM-Based Cytokeratin Marker Assay as a Tool for Cancer Studies: Application to Lung Cancer Pleural Effusions." Proteomics Clin Appl **12**(2).
Planque, C., V. Kulasingam, C. R. Smith, K. Reckamp, L. Goodglick and E. P. Diamandis (2009). "Identification of Five Candidate Lung Cancer Biomarkers by Proteomics Analysis of Conditioned Media of Four Lung Cancer Cell Lines." MOLECULAR & CELLULAR PROTEOMICS **8**(12): 2746-2758.
Poschmann, G., A. Lendzian, J. Uszkoreit, M. Eisenacher, A. V. Borght, F. C. Ramaekers, H. E. Meyer and K. Stühler (2013). "A combination of two electrophoretical approaches for detailed proteome-based characterization of SCLC subtypes." Arch Physiol Biochem **119**(3): 114-125.
Prabhu, A., K. Scott, P. Stewart, D. Grass, M. Fernandez, J. Koomen, T. Bannister, S. Sumner, C. Rudin, G. Denicola, J. Cleveland and E. Haura (2020). "Integrated Proteometabolomic Analysis Reveals Metabolic Vulnerabilities in Small-Cell Lung Cancer." JOURNAL OF THORACIC ONCOLOGY **15**(2): S19-S19.
Rajasekaran, S., J. Siddiqui, J. Rakijas, B. Nicolay, C. Y. Lin, E. Khan, R. Patel, R. Morris, E. Wyler, M. Boukhali, J. Balasubramanyam, R. R. Kumar, C. Van Rechem, C. Vogel, S. V. Elchuri, M. Landthaler, B. Obermayer, W. Haas, N. Dyson and W. Miles (2021). "Integrated multi-omics analysis of RB-loss identifies widespread cellular programming and synthetic weaknesses." COMMUNICATIONS BIOLOGY **4**(1).
Rikova, K., B. Hall, T. Levy, A. Possemato, M. Aguiar, S. Beausoleil, J. M. Ren, K. L. Lee, S. Lonning and M. Comb (2016). "Proteomic analysis identifies multi-dimensional deregulated signaling pathways in SCLC lung cancer." CANCER RESEARCH **76**.
Rosell, R. and L. Wannesson (2012). "A genetic snapshot of small cell lung cancer." Cancer Discov **2**(9): 769-771.
Seike, M., T. Kondo, K. Fujii, T. Okano, T. Yamada, Y. Matsuno, A. Gemma, S. Kudoh and S. Hirohashi (2005). "Proteomic signatures for histological types of lung cancer." Proteomics **5**(11): 2939-2948.
Sen, T., P. Tong, L. X. Diao, L. R. Li, Y. H. Fan, J. Hoff, J. V. Heymach, J. Wang and L. A. Byers (2017). "Targeting AXL and mTOR Pathway Overcomes Primary and Acquired Resistance to WEE1 Inhibition in Small-Cell Lung Cancer." CLINICAL CANCER RESEARCH **23**(20): 6239-6253.
Sen, T., P. Tong, C. A. Stewart, S. Cristea, A. Valliani, D. S. Shames, A. B. Redwood, Y. H. Fan, L. R. Li, B. S. Glisson, J. D. Minna, J. Sage, D. L. Gibbons, H. Piwnica-Worms, J. V. Heymach, J. Wang and L. A. Byers (2017). "CHK1 Inhibition in Small-Cell Lung Cancer Produces Single-Agent Activity in Biomarker-Defined Disease Subsets and Combination Activity with Cisplatin or Olaparib." CANCER RESEARCH **77**(14): 3870-3884.

Sen, T., P. Tong, J. Wang and L. A. Byers (2016). "Proteomic profiling identifies ATM expression level as a predictive biomarker to ATR and PARP inhibition in small cell lung cancer (SCLC)." CANCER RESEARCH **76**.
Stewart, C. A., P. Tong, R. J. Cardnell, T. Sen, L. Li, C. M. Gay, F. Masrorpour, Y. Fan, R. O. Bara, Y. Feng, Y. Ru, J. Fujimoto, S. T. Kundu, L. E. Post, K. Yu, Y. Shen, B. S. Glisson, I. Wistuba, J. V. Heymach, D. L. Gibbons, J. Wang and L. A. Byers (2017). "Dynamic variations in epithelial-to-mesenchymal transition (EMT), ATM, and SLFN11 govern response to PARP inhibitors and cisplatin in small cell lung cancer." Oncotarget **8**(17): 28575-28587.
Stewart, P. A., J. N. Li, K. J. Fisher, S. Dhungana, D. Stewart, S. Sumner, E. Gardner, J. Poirier, C. M. Rudin, E. A. Welsh, S. Eschrich, A. Chen and E. B. Haura (2015). "Integrating proteomics and metabolomics characterizes active pathways and potential drug targets in small cell lung cancer." CANCER RESEARCH **75**.
Sumi, N. J., C. Ctortecka, Q. Hu, A. T. Bryant, B. Fang, L. L. Remsing Rix, M. Ayaz, F. Kinose, E. A. Welsh, S. A. Eschrich, H. R. Lawrence, J. M. Koomen, E. B. Haura and U. Rix (2019). "Divergent Polypharmacology-Driven Cellular Activity of Structurally Similar Multi-Kinase Inhibitors through Cumulative Effects on Individual Targets." Cell Chemical Biology **26**(9): 1240-1252.e1211.
Szeitz, B., Z. Megyesfalvi, N. Woldmar, Z. Valkó, A. Schwendenwein, N. Bárány, S. Paku, V. László, H. Kiss, E. Bugyik, C. Lang, A. M. Szász, L. Pizzatti, K. Bogos, M. A. Hoda, K. Hoetzenecker, G. Marko-Varga, P. Horvatovich, B. Döme, K. Schelch and M. Rezeli (2022). "In-depth proteomic analysis reveals unique subtype-specific signatures in human small-cell lung cancer." Clin Transl Med **12**(9): e1060.
Tanca, A., M. F. Addis, D. Pagnozzi, P. Cossu-Rocca, R. Tonelli, G. Falchi, A. Eccher, T. Roggio, G. Fanciulli and S. Uzzau (2011). "Proteomic analysis of formalin-fixed, paraffin-embedded lung neuroendocrine tumor samples from hospital archives." JOURNAL OF PROTEOMICS **74**(3): 359-370.
Togayachi, A., J. Iwaki, H. Kaji, H. Matsuzaki, A. Kuno, Y. Hirao, M. Nornura, M. Noguchi, Y. Ikehara and H. Narimatsu (2017). "Glycobiomarker, Fucosylated Short-Form Secretogranin III Levels Are Increased in Serum of Patients with Small Cell Lung Carcinoma." JOURNAL OF PROTEOME RESEARCH **16**(12): 4495-4505.
Toyokawa, G., M. Kodama, N. Haratake, Y. Yamada, H. Kittaka, T. Takenaka, K. Tanaka, M. Shimokawa, K. Yamazaki, S. Takeo, I. Okamoto, Y. Oda and K. I. Nakayama (2021). "Comprehensive analysis of the metabolic enzymes in patients with small cell lung cancer using a large-scale targeted proteomics assay." ANNALS OF ONCOLOGY **32**: S1169-S1169.
Tripathi, S. C., J. F. Fahrmann, M. Celiktas, M. Aguilar, K. D. Marini, M. K. Jolly, H. Katayama, H. Wang, E. N. Murage, J. B. Dennison, D. N. Watkins, H. Levine, E. J. Ostrin, A. Taguchi and S. M. Hanash (2017). "MCAM Mediates Chemoresistance in Small-Cell Lung Cancer via the PI3K/AKT/SOX2 Signaling Pathway." CANCER RESEARCH **77**(16): 4414-4425.
Udyavar, A. R., M. D. Hoeksema, J. E. Clark, Y. Zou, Z. Tang, Z. Li, M. Li, H. Chen, A. Statnikov, Y. Shyr, D. C. Liebler, J. Field, R. Eisenberg, L. Estrada, P. P. Massion and V. Quaranta (2013). "Co-expression network analysis identifies Spleen Tyrosine Kinase (SYK) as a candidate oncogenic driver in a subset of small-cell lung cancer." BMC SYSTEMS BIOLOGY **7**.
Villalobos-Manzo, R., E. Ríos-Castro, J. M. Hernández-Hernández, G. Oza, M. A. Medina and J. Tapia-Ramírez (2022). "Identification of Transferrin Receptor 1 (TfR1) Overexpressed in Lung Cancer Cells, and Internalization of Magnetic Au-CoFe(2)O(4) Core-Shell Nanoparticles Functionalized with Its Ligand in a Cellular Model of Small Cell Lung Cancer (SCLC)." Pharmaceutics **14**(8).
Wang, X., Y. Zhang, C. L. Nilsson, F. S. Berven, P. E. Andrén, E. Carlsohn, P. Horvatovich, J. Malm, M. Fuentes, Á. Végvári, C. Welinder, T. E. Fehniger, M. Rezeli, G. Edula, S. Hober, T. Nishimura and G. Marko-Varga (2015). "Association of chromosome 19 to lung cancer genotypes and phenotypes." Cancer and Metastasis Reviews **34**(2): 217-226.
Wu, J. Y., Z. F. Hao, C. Ma, P. F. Li, L. Y. Dang and S. S. Sun (2020). "Comparative proteogenomics profiling of non-small and small lung carcinoma cell lines using mass spectrometry." PEERJ **8**.

Xiao, H., L. Zhang, H. Zhou, J. M. Lee, E. B. Garon and D. T. Wong (2012). "Proteomic analysis of human saliva from lung cancer patients using two-dimensional difference gel electrophoresis and mass spectrometry." Mol Cell Proteomics **11**(2): M111.012112.
Yang, R. H., R. F. Tian, Q. L. Ren, H. Y. Chui, S. T. Guo, X. D. Zhang and X. Song (2016). "Serum protein profiles of patients with lung cancer of different histological types." Asia Pac J Clin Oncol **12**(1): 70-76.
Yang, S., W. Zhang, B. Zhou, Y. Tian, Y. Nan, L. Bu, Y. Ruan, X. Sun and D. Yang (2006). "Analysis of differential expression proteins of small cell lung cancer and matched normal lung tissues by 2-DE and MALDI-TOF-MS." Journal of Xi'an Jiaotong University (Medical Sciences) **27**(6): 533-537.
Yang, S. Y., X. Y. Xiao, W. G. Zhang, L. J. Zhang, W. Zhang, B. Zhou, G. Chen and D. C. He (2005). "Application of serum SELDI proteomic patterns in diagnosis of lung cancer." BMC CANCER **5**.
Yin, Y. P., W. H. Shi, K. Deng, X. L. Liu, H. Li, X. T. Lv, V. W. Y. Lui, C. Ding, B. Hong and W. C. Lin (2021). "Combinations of proteasome inhibitors with obatoclax are effective for small cell lung cancer." ACTA PHARMACOLOGICA SINICA **42**(8): 1298-1310.
Yoshimura, H., Y. Takeda, T. Koba and A. Kumanogoh (2021). "Discovery of Novel Biomarkers of Small Cell Lung Cancer by Proteomics of Exosomes." AMERICAN JOURNAL OF RESPIRATORY AND CRITICAL CARE MEDICINE **203**(9).
Yu, L., K. Li, Z. Xu, G. Cui and X. Zhang (2018). "Integrated omics and gene expression analysis identifies the loss of metabolite-metabolite correlations in small cell lung cancer." Onco Targets Ther **11**: 3919-3929.
Yurdakul, A. S., O. Sonmez, C. Simsek, N. Zengin, A. I. Keyf, C. Ozturk, O. B. Oksuzoglu, D. Kubilay, O. Z. Gulbahar, S. C. O. Karatayli and A. M. Bozdayi (2012). "Evaluation of Relationship Between Serum Protein Profiles and Lung Cancer by SELDI-TOF-MS (Surface Enhanced Laser Desabsorbtion Ionization Time of Flight Mass Spectrometry) Method." TURKIYE KLINIKLERI TIP BILIMLERI DERGISI **32**(4): 1084-1089.
Zhou, Y. Y., T. M. Lih, J. B. Pan, N. Hoti, M. M. Dong, L. W. Cao, Y. W. Hu, K. C. Cho, S. Y. Chen, R. V. Eguez, E. Gabrielson, D. W. Chan, H. Zhang and Q. K. Li (2020). "Proteomic signatures of 16 major types of human cancer reveal universal and cancer-type-specific proteins for the identification of potential therapeutic targets." JOURNAL OF HEMATOLOGY & ONCOLOGY **13**(1).
Ziv, T., E. Barnea, H. Segal, R. Sharon, I. Beer and A. Admon (2006). "Comparative proteomics of small cell lung carcinoma." Cancer Biomark **2**(6): 219-234.
